# Understanding fundamental principles of enhancer biology at a model locus: Analysing the structure and function of an enhancer cluster at the α‐globin locus

**DOI:** 10.1002/bies.202300047

**Published:** 2023-07-05

**Authors:** Mira Kassouf, Seren Ford, Joseph Blayney, Doug Higgs

**Affiliations:** ^1^ Laboratory of Gene Regulation MRC Weatherall Institute of Molecular Medicine Radcliffe Department of Medicine University of Oxford Oxford UK

**Keywords:** alpha globin locus, enhancer cluster, enhancer function, enhanceropathies, erythropoiesis, gene regulation, super‐enhancer

## Abstract

Despite ever‐increasing accumulation of genomic data, the fundamental question of how individual genes are switched on during development, lineage‐specification and differentiation is not fully answered. It is widely accepted that this involves the interaction between at least three fundamental regulatory elements: enhancers, promoters and insulators. Enhancers contain transcription factor binding sites which are bound by transcription factors (TFs) and co‐factors expressed during cell fate decisions and maintain imposed patterns of activation, at least in part, via their epigenetic modification. This information is transferred from enhancers to their cognate promoters often by coming into close physical proximity to form a ‘transcriptional hub’ containing a high concentration of TFs and co‐factors. The mechanisms underlying these stages of transcriptional activation are not fully explained. This review focuses on how enhancers and promoters are activated during differentiation and how multiple enhancers work together to regulate gene expression. We illustrate the currently understood principles of how mammalian enhancers work and how they may be perturbed in enhanceropathies using expression of the α‐globin gene cluster during erythropoiesis, as a model.

## Graphical abstract

Events leading to gene activation during differentiation include binding of key transcription factors (TFs) and co‐factors; loop extrusion juxtaposing enhancers and promoters, and finally recruitment of Pol II to initiate transcription. Gene expression is controlled by clusters of enhancer‐like elements including activators and facilitators. Such clusters may act in an orientation‐dependent manner.

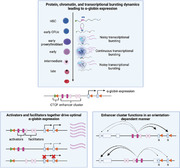

**Abbreviations**
ATAC‐seq: Assay for Transposase‐Accessible Chromatin using sequencing
ChIP‐seq: Chromatin Immunoprecipitation followed by sequencing
CTCF: CCCTC‐binding factor
eRNA: enhancer‐RNA
LCR: locus control region
PIC: pre‐initiation complex
Pol II: RNA polymerase II
SE: super‐enhancer
TAD: topologically associating domain
TFs: transcription factors

## INTRODUCTION

It is estimated that in mammalian genomes there are ∼ 20 000 genes regulated by ∼900 000 enhancer‐like elements,^[^ ^1^ ^]^ interspersed with ∼30 000 CCCTC‐binding factor (CTCF)‐bound elements,^[^ ^2^, ^3^ ^]^ many of which act as insulators. The genome encodes > 100 000 RNAs, which produce over 400 000 proteins. Within the context of chromatin, genomic elements, RNA and proteins can be modified in a manner that alters their function. Over the past two decades, the development of new sequencing and proteomic technologies have enabled us to accurately document all of these phenomena in populations of cells and increasingly, in single cells. New computational tools, including the use of artificial intelligence and mathematical modelling, allow us to search for or probe patterns that are starting to reveal common principles of gene regulation, how networks are established and how they interact. Importantly, we are also documenting how these processes are perturbed in human genetic disease. Despite these huge advances and the availability of an almost overwhelming resource, we still do not fully understand the mechanisms by which individual genes are switched on or off during development, lineage specification and differentiation. In our work, we have focussed on understanding the regulation of a single gene using the orthologous human and mouse α‐globin loci as our model and this continues to provide new insights into how enhancers control gene expression in the context of a regulatory domain.

**FIGURE 1:**
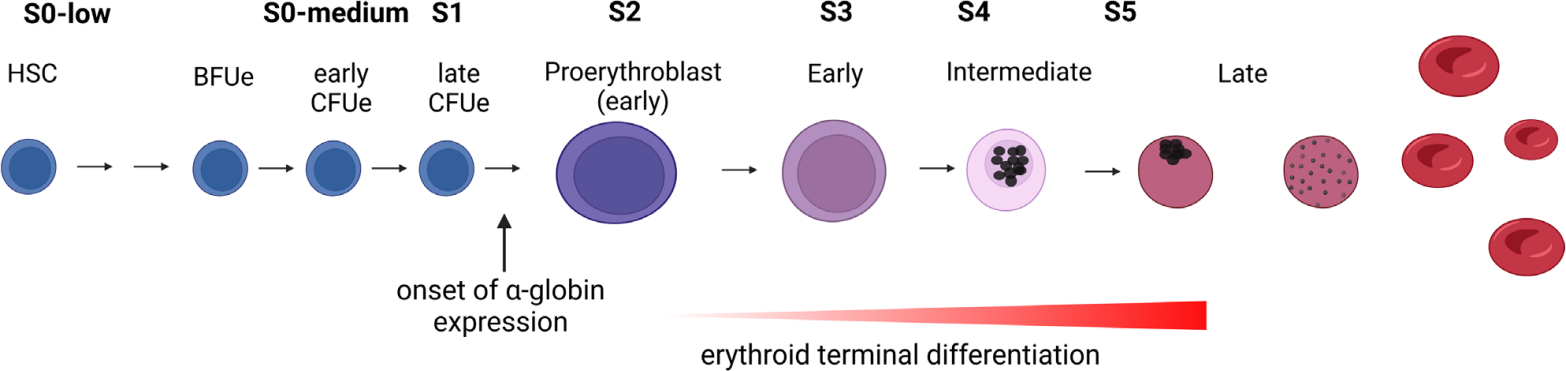
Schematic representation of erythropoiesis. S0‐low to S5 mark seven defined cellular stages of erythropoiesis representing an immunophenotyping‐based purification strategy^[^ ^7^ ^]^ that allows the isolation of the desired population from mouse fetal livers. The stages of erythroid differentiation are shown starting with Hematopoietic Stem Cells (HSC) committing, amongst other lineages, to the erythroid progenitors (burst colony forming unit and colony forming unit erythroid, BFUe and CFUe respectively). α‐globin expression is first detected and progressively increases as cells differentiate from proerythroblasts to mature red blood cells, as indicated by the gradually increasing red colour in the red triangle. The schematic representation of the progressively maturing erythroid cells highlight the morphologically distinguishable stages both in the varying size and hemoglobinisation states of the cells.

The globin genes are exclusively expressed during the process of erythropoiesis, which produces 1–2 million red blood cells every second in healthy adults^[^ ^4^ ^]^ and occurs when haematopoietic stem cells undergo lineage specification and differentiation (Figure 1). The process of erythropoiesis is very well understood and cells at various stages can be purified to obtain sequential snapshots as the locus is activated and the genes are ultimately transcribed.^[^ ^5^, ^6^, ^7^ ^]^

Mutations of the human α‐globin locus cause a common form of inherited anaemia (α‐thalassaemia) and therefore provide a rich source of informative naturally occurring mutations including mutations of the enhancer cluster.^[^ ^8^ ^]^ Such mutations can be modelled in the orthologous mouse locus, which faithfully recapitulates the molecular and cellular phenotypes arising from human mutations. The mouse also provides an excellent test‐bed for producing newly engineered mutations, not present in humans, to further test hypotheses concerning gene regulation. Importantly, unlike many other genes that have been analysed, α‐globin has no downstream targets and therefore during erythropoiesis, natural and engineered mutations do not alter the expression of any other genes that might affect cell fate and confound the primary effects of the mutations that are introduced. This is an ideal situation when analysing the principles by which enhancers regulate transcription rather than how they control cell fate.

It is often argued that understanding a single locus might not reveal the general principles of gene regulation: evolution is only influenced by the selective advantage of the final output and so there may be great differences in the mechanisms of mammalian gene regulation. Whilst this is possible, all mechanisms of gene regulation first established at the α‐ and β‐globin loci have been found to be very widely used. These include nearly all processes involving enhancer‐driven expression, insulator elements, transcription, RNA processing and translation. We are therefore optimistic that the mechanisms by which the globin enhancers communicate with and activate transcription from their cognate promoters will elucidate general principles of gene regulation.

Here we review current information about the order of events as the mouse α‐globin enhancers and promoters are activated during erythropoiesis. We will then focus on two important unanswered questions in enhancer biology. First, what are the roles of individual elements in the context of a cluster of enhancers (sometimes referred to as a locus control region [LCR] or super‐enhancer [SE]): do the enhancer elements act individually or as a group? Second, in contrast to individual enhancers, do enhancer clusters act in an orientation‐dependent manner? Finally, we will discuss our current understanding of how the enhancer communicates with the promoter and how this process is perturbed in enhanceropathies.

## THE STRUCTURE OF THE α‐GLOBIN CLUSTER

**FIGURE 2:**
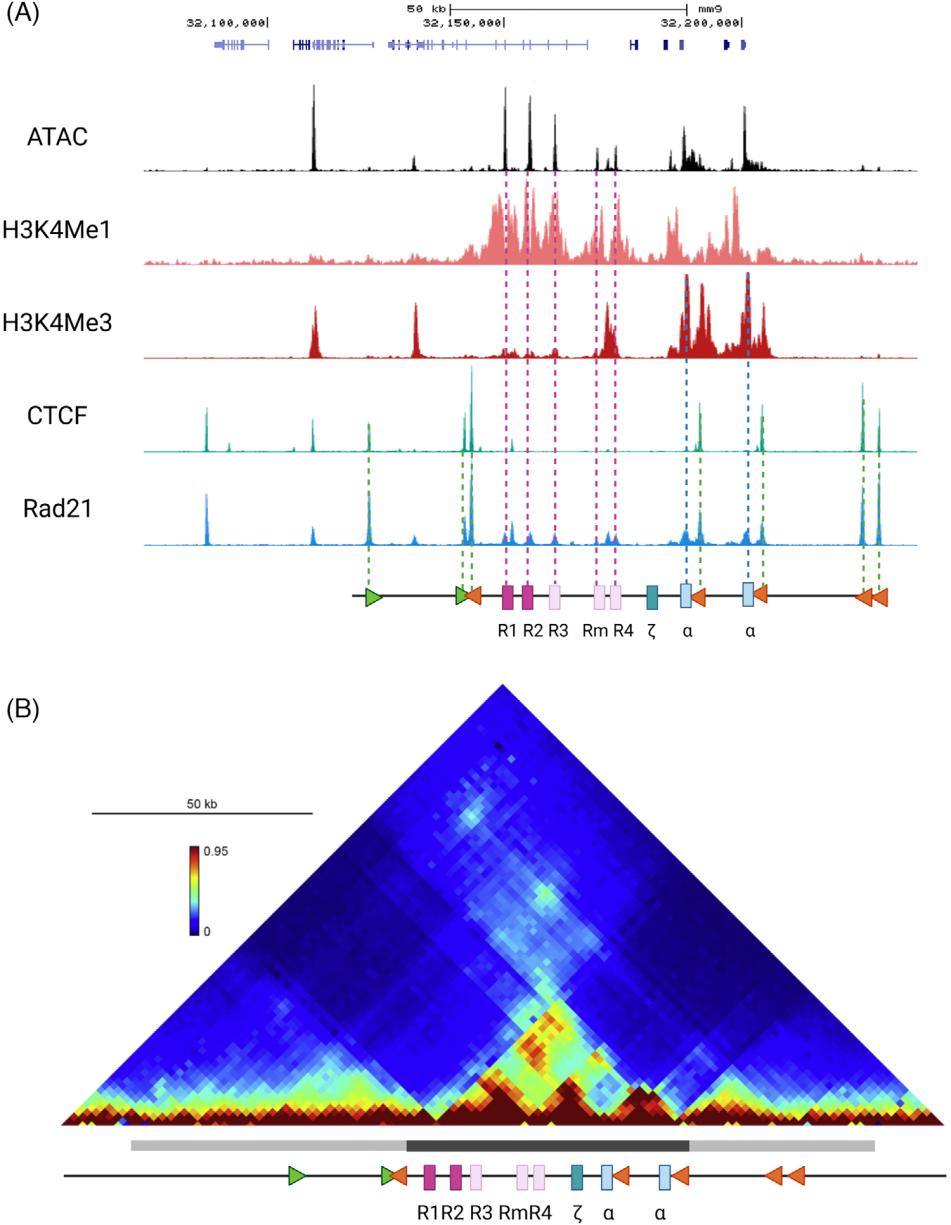
The α‐globin locus regulatory domain. (A) At the top, UCSC track representing the α‐globin locus (coordinates [mm9]: 32 120 000–32 200 000). Middle, the regulatory elements of the α‐globin locus are indicated by accessible chromatin (Assay for Transposase‐Accessible Chromatin using sequencing: ATAC‐seq) and Chromatin Immunoprecipitation followed by sequencing (ChIP‐seq) tracks for H3K4me1 marking the enhancers (dark and light pink rectangles), H3K4me3 indicating the promoters (light blue rectangles), CTCF binding pattern corresponding to the CTCF sites (green and orange triangles), and Rad21 binding across the locus. Bottom, a schematic representation of the locus with the elements represented as described above and specifically the enhancers R1 and R2 and facilitators R3, Rm and R4 as well as the embryonic (ζ) and adult α‐globin genes indicated. (B) Chromatin conformation capture (3C, Tiled‐C Capture) contact matrix covering 200 kb spanning the mouse α‐globin cluster (coordinates [mm9]: 32 060 000–32 260 000) with the higher intensity colours reflecting the higher frequency of contact between the α‐globin major cis‐regulatory elements and delineating the sub‐TAD (dark grey bar under the matrix), and contacts established between the convergent CTCF sites marking the span of the TAD (light grey bar under the matrix), both structures aligned to the schematic of the locus below.

The mouse α‐globin cluster on chromosome 11, is located in a ∼65 kb erythroid‐specific sub‐TAD (topologically associating domain), which is contained within a larger ∼165 kb TAD present in all tested cell types (Figure 2),^[^ ^9^ ^]^ typical structures emerging as a consequence of loop‐extrusion delimited by largely convergent CTCF boundary elements ^[^ ^10^ ^]^ and reflecting domains of preferred interactions amongst DNA elements.^[^ ^11^, ^12^ ^]^ The locus includes an embryonic ζ‐globin gene (Hba‐x) and a pair of almost identical adult α‐globin genes (Hba‐1 and Hba‐2). The cluster also contains two θ‐globin genes (Hbθ‐1 and Hbθ‐2) of unknown function.

All of these genes are regulated by a set of five erythroid‐specific enhancer elements (R1, R2, R3, Rm and R4) present ∼8–31 kb 5′ (upstream) of the cluster (Figure 2A). Four of these enhancers (R1, R2, R3 and Rm) are located in introns of the adjacent widely expressed gene Nprl3. Each enhancer contains various combinations of binding sites for TFs known to regulate erythropoiesis (GATA1, TAL1, NFE2 and KLF1) and the enhancer cluster fulfils the definition of a SE.^[^ ^13^, ^14^ ^]^ The α‐globin genes and their enhancers are flanked by multiple largely convergent CTCF‐binding elements at the boundaries of the sub‐TAD.^[^ ^15^ ^]^ These elements in the mouse are largely conserved in the human α‐globin locus.^[^ ^16^ ^]^

## THE CELLULAR EVENTS IN ERYTHROPOIESIS

Globin gene expression occurs within the context of erythropoiesis (Figure 1). Via different pathways, haematopoietic stem cells ultimately form bi‐potential progenitors which may differentiate into erythroid cells or megakaryocytes which eventually form platelets required for haemostasis. The first cell type fully committed to erythropoiesis alone is referred to as the erythroid burst forming unit (BFU‐E), retains considerable capacity for expansion and gives rise to erythroid colony forming units (CFU‐Es). Of interest, these cells pass through a cell cycle with an unusually rapid S‐phase^[^ ^7^ ^]^; they then become fully committed to terminal differentiation and form a synchronous population of cells which progress through 3–4 subsequent divisions and ultimately enucleate to form mature red blood cells. Using flow cytometry, in mice these cells have been stratified into seven subgroups (S0 low and S0 medium, S1, S2, S3, S4 and S5)^[^ ^7^ ^]^ (Figure 1). Using single‐cell RNA‐seq, α‐globin RNA can be detected at basal levels even in stem and progenitor cells (S0).^[^ ^9^ ^]^ Expression of α‐globin increases dramatically as cells transition from late CFU‐E (S1) to proerythroblasts (S2), and plateaus at S3 as cells become fully committed to terminal differentiation.^[^ ^9^, ^17^ ^]^

## THE ORDER OF EVENTS LEADING TO α‐GLOBIN TRANSCRIPTION

Using primary erythroid cells and erythroid cell lines corresponding to the various stages of mouse erythropoiesis (S0–S5), the order of events leading to activation of the α‐globin genes has been characterised in some detail.^[^ ^9^, ^17^ ^]^ It is important to consider these events not as fixed step‐wise progressions; rather they are highly dynamic processes by which the probability of activation increases with time throughout differentiation.

**FIGURE 3:**
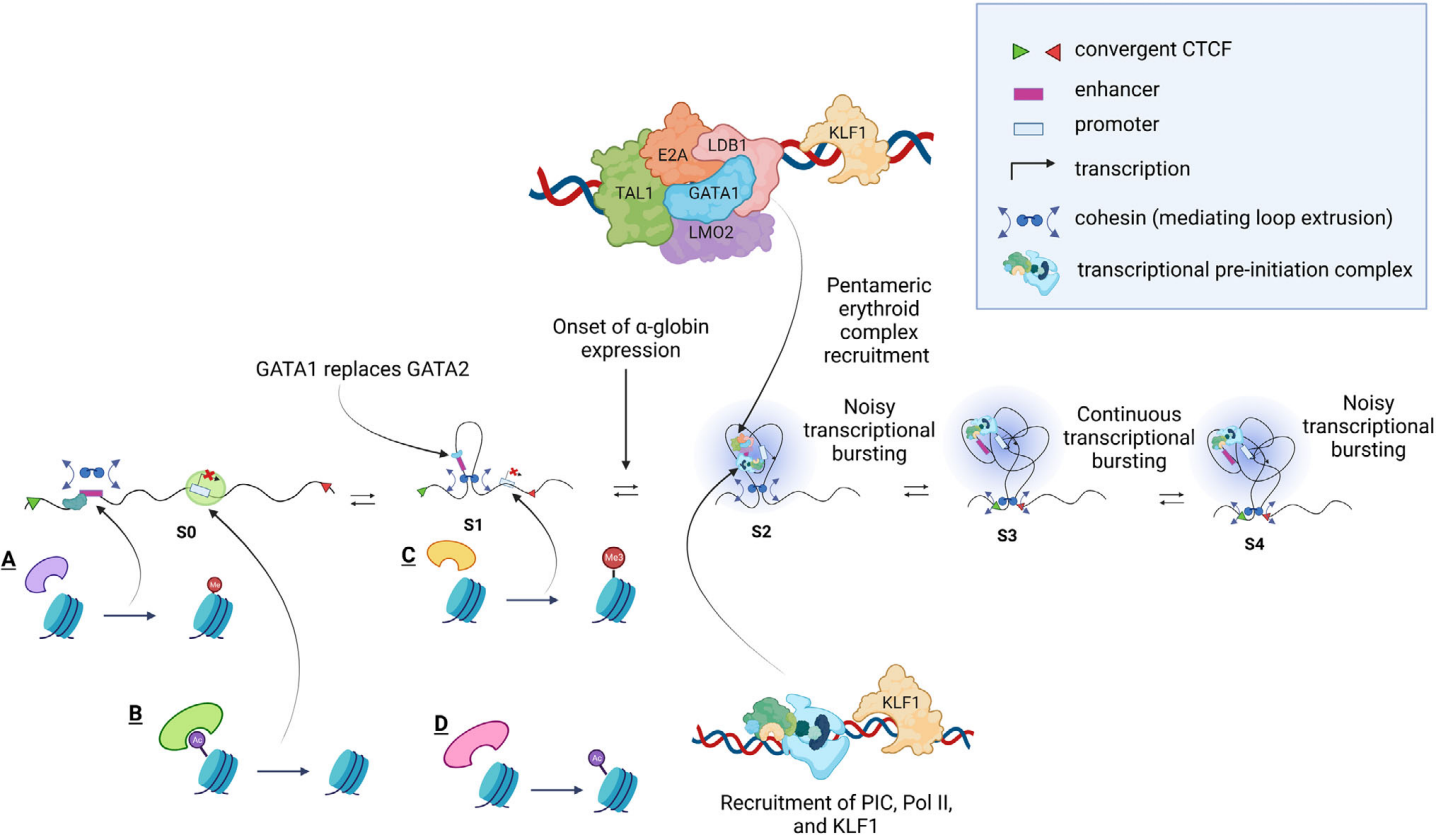
The dynamics of the molecular events at the α‐globin locus throughout the concomitant erythroid differentiation and α‐globin expression. The series of events from early erythroid progenitors (S0) where GATA2 acts as a pioneer factor at enhancers, low contact frequency between α‐globin enhancers and promoters, α‐globin is not transcribed (A—H3K4me1 at enhancers deposited by MLL3/4 COMPASS, B—Polycomb‐mediated repression of gene expression at promoters). As the erythroid differentiation progresses (S1), GATA1 replaces GATA2 (C—H3K4me3 deposited at promoters by the trithorax proteins MLL1 and MLL2, D—Polycomb complex removed and high levels of H3K27Ac deposited by p300 and CBP leading to maximum chromatin accessibility). The more differentiated erythroid progenitor state (S2) is marked by a more established binding of erythroid‐specific transcription factors (the pentameric complex) and by an increase in contact probability between the enhancer and promoters and noisy transcriptional bursting. As the more mature erythroid cells emerge (S3), continuous transcriptional bursting is observed, followed by a return to a noisy transcriptional bursting state (S4).^[^ ^73^ ^]^

Changes in α‐globin expression are driven by changes in the protein factors bound at the α‐globin enhancers and promoters, whilst insulator elements are bound by CTCF at all stages of erythropoiesis. In early progenitors and precursors, the α‐globin gene promoters, but not the enhancers, are bound by components of the polycomb complex (Figure 3) which is thought to maintain the silencing of these genes, at least in part, via histone deacetylation.^[^ ^18^ ^]^ This process was originally identified at the human α‐globin cluster but has more recently been shown to also occur at the mouse locus (Beagrie R et al., in preparation). During activation of the enhancers, the polycomb complex and H3K27me3 are removed, probably influenced by the histone demethylase JMJD3,^[^ ^19^ ^]^ a component of the MLL3 and MLL4 COMPASS complex in mammals.^[^ ^20^, ^21^, ^22^ ^]^

In early erythroid cells, the α‐globin regulatory elements are also bound by GATA2 suggesting it may act as a pioneer factor^[^ ^23^ ^]^ for opening chromatin. In early progenitors, low levels of other TFs involved in α‐globin expression (GATA1, TAL1, LDB1 and ZBP89) are also found at the enhancer elements^[^ ^24^, ^25^ ^]^ (Figure 3). In these precursors, enhancers are also modified by H3K4me1, implying that the MLL3/4 COMPASS complex is also present and active at these sites.^[^ ^21^, ^22^, ^26^ ^]^ At these early stages of erythropoiesis, when the α‐genes are not being transcribed the level of histone acetylation is low and contact frequency between the α‐globin enhancers and promoters appears relatively low.^[^ ^9^ ^]^ The appearance of enhancer‐RNAs (eRNAs) at enhancers precedes the activation of nearby genes; the occurrence of eRNAs has been variously proposed to keep the enhancer region in an open configuration,^[^ ^27^, ^28^ ^]^ and/or to facilitate enhancer‐promoter looping^[^ ^29^, ^30^ ^]^ (eRNA roles are extensively reviewed in ref.[31]). Together, these findings suggest that in the stages before the α‐genes are transcribed (particularly S0 low and S0 medium), the regulatory elements are forming via a dynamic process while the target genes are substantially repressed via the polycomb complex, preventing premature activation of the α‐genes.

Several changes occur at the critical stage of erythroid commitment (S1–S2). The polycomb complex is removed. Chromatin accessibility reaches its maximum level. Histone acetylation is thought to occur via P300 and CBP.^[^ ^22^, ^32^, ^33^, ^34^ ^]^ GATA1 is recruited at high levels fully replacing GATA2. This ‘GATA switch’, crucial for progressive erythroid maturation, reflects the RNA levels of these TFs and is thought to be driven by the increasing abundance of GATA1 displacing the decreasing levels of GATA2.^[^ ^35^ ^]^ However, this interpretation of the GATA switch was questioned by a report showing discrepancy between the levels of GATA1/2 RNAs and proteins as differentiation proceeds.^[^ ^36^ ^]^ The CCAAT‐box binding factor (NFY) is detectable at the promoters and the levels of H3K4me1 (at enhancers) and H3K4me3 (at promoters) mediated by the Histone (H) Lysine (K) methyltransferarses, MLL3/4 and MLL1/2, respectively, reach their maximum levels. However, at this stage, there are still no readily detectable components of the pre‐initiation complex (PIC) or RNA polymerase II (Pol II) at the α‐globin promoters. Thus, it appears that, at this stage of erythropoiesis, the elements are ready for activation but relatively little α‐globin transcription occurs (Figure 3).

Activation of α‐globin expression occurs at the cell transition between S1 and S2 reaching a maximum at S2–S3. This activation is associated with recruitment of TAL1 (at the enhancers) possibly as a member of the pentameric erythroid complex (TAL1, E2A, LDB1, LMO2 and GATA1).^[^ ^25^, ^37^, ^38^ ^]^ Activation is also associated with recruitment of KLF1 at the enhancers and promoters.^[^ ^39^, ^40^ ^]^ The recruitment of all tested components of the PIC and Pol II to the α‐globin promoters is documented^[^ ^39^ ^]^ as well as an increase in histone acetylation across most of the sub‐TAD. It appears that the primary role of the enhancers is to recruit the PIC and initiate transcription.^[^ ^41^ ^]^ Although this is the primary effect, it does not rule out a role for enhancers in subsequent stages of the transcription cycle. Increased α‐globin expression is associated with a concomitant increase in contact probability between the enhancer and promoter.^[^ ^9^ ^]^

It is clear that the Mediator complex and its co‐factor BRD4 play a role in activating α‐globin transcription as both protein complexes are present at the α‐globin enhancers and promoters in committed erythroid cells^[^ ^14^ ^]^ and reduced when the enhancers and α‐globin transcription are compromised.^[^ ^42^ ^]^ Of interest, the Mediator complex has been shown to occupy enhancers in the β‐globin LCR in ES cells, even though looping of this enhancer with its promoter does not occur until the erythroid lineage.^[^ ^43^, ^44^ ^]^ In future, to complete the model, it will be important to determine exactly when the Mediator complex and BRD4 complexes are recruited to the α‐globin enhancers during erythropoiesis.

The current model suggests that the enhancers are first primed, then interact with the promoter and facilitate recruitment of the PIC and Pol II. This is supported by human enhanceropathies which cause α‐thalassaemia by deletion of the enhancers and experiments in mice in which the enhancers have been partially or completely deleted. In all cases, the primary effect of these mutations is to reduce the recruitment of the PIC and Pol II (see below).

## HOW ARE INTERACTIONS BETWEEN ENHANCERS AND PROMOTERS ESTABLISHED DURING DIFFERENTIATION?

The 165 kb TAD containing the α‐globin cluster and five widely expressed genes lying upstream is found in all cell types tested. By contrast, the 65 kb sub‐TAD containing the α‐genes and their enhancers is only seen in erythroid cells.^[^ ^9^ ^]^ Within single cells analysed by Hi‐C, defined fixed TADs do not exist: it is only when populations of cells are considered that the patterns of TADs emerge.^[^ ^45^, ^46^ ^]^ Therefore, it is important to consider TADs and sub‐TADs as interaction probabilities rather than as defined structures. Similarly, interactions which juxtapose enhancers and promoters within sub‐TADs are dynamic with estimated time spans of 15 min^[^ ^47^, ^48^ ^]^ The true rate of molecular contact between enhancers and promoters at the nanoscale is challenging to study because of technical limitations.^[^ ^49^ ^]^ Of interest, the sub‐TAD containing the α‐globin locus defined by imaging can be found in 76% of erythroid cells^[^ ^50^ ^]^ although the precise borders defining these structures are not known at high resolution since the probes cover relatively large regions (64–139 kb).^[^ ^50^ ^]^

**FIGURE 4:**
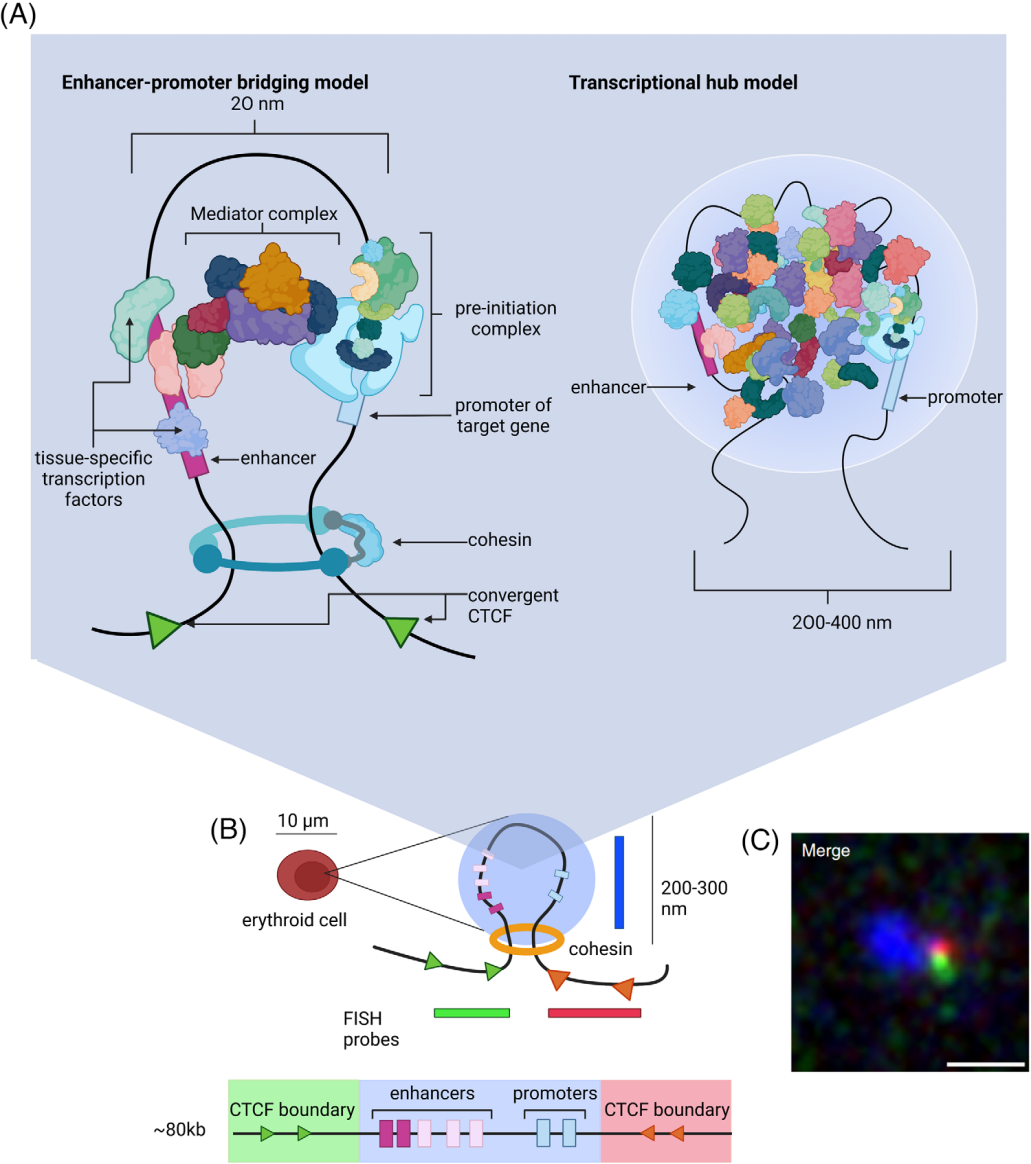
Theoretical and α‐globin‐specific depiction of enhancer‐promoter interactions. (A) Two models describing molecular states of enhancer‐promoter interaction. In both models, it is thought that proximity between the enhancer and promoter may be, at least in part, the result of loop extrusion. How information is transferred from the enhancer to the promoter is unknown. One possibility is that the Mediator complex provides a protein bridge between the two elements. Another model proposes the formation of a transcriptional hub in which molecular crowding or liquid‐liquid phase separation forms a high concentration of TFs and CoFs creating an environment that is conducive to transcription. (B) A schematic representation of the α‐globin interacting domain, as visualised using super‐resolution microscopy, formed specifically in erythroid cells whereby enhancers and promoters are interacting in a de‐compacted segment of chromatin delimited by flanking convergent CTCF sites. Below, a linear representation of the locus with all the elements indicated as in Figure 2, and highlighting in colour the segments that correspond to probes used in the labelling process; green and red for the flanks and blue for the enhancer‐promoter region. The schematic above shows the enhancer‐promoter interaction domain observed only in erythroid cells. (C) The super‐resolution image of the α‐globin interaction domain; the blue probe highlights de‐compacted chromatin spanning the α‐globin enhancers and promoters bounded by the flanking red and green probes encompassing the convergent CTCF sites. Scale bar 0.5 μm.

Reconciling the chromatin structure models inferred from chromatin conformation capture population averages (3C and Hi‐C) and single‐cell imaging approaches remains a challenge.^[^ ^51^, ^52^ ^]^ The view of chromatin organisation as a dynamic ensemble of contacts impacts discussions around enhancer‐promoter interactions, especially around how stable or transient they are.^[^ ^52^ ^]^ What brings the elements into close proximity is also a highly debatable topic.^[^ ^53^, ^54^ ^]^ Popular models explaining enhancer‐promoter contacts involve loop extrusion^[^ ^10^, ^55^, ^56^ ^]^ and/or passive diffusion of enhancers and promoters.^[^ ^57^, ^58^ ^]^ These mechanisms are not mutually exclusive. Several lines of evidence suggest that both TADs and sub‐TADs are formed by loop extrusion (Figure 4A), itself a dynamic process, mediated by the cohesin complex and delimited by CTCF‐bound insulators. Consistent with this, both cohesin and its associated protein NIPBL, which is thought to load cohesin and play a role in its translocation,^[^ ^59^ ^]^ are detectable at increased levels at the α‐globin enhancers and promoters in erythroid cells.^[^ ^15^, ^60^ ^]^ Furthermore, deletion of two CTCF‐bound insulators lying upstream of the α‐globin enhancers extends the sub‐TAD and this is associated with activation of newly incorporated genes within the extended sub‐TAD.^[^ ^15^ ^]^ It is also possible that enhancer‐promoter proximity occurs by passive diffusion (Figure 4A). However proximity is induced, contacts are thought to be transiently stabilised by homotypic protein interactions, for example involving the LDB1 component of the erythroid pentameric complex^[^ ^61^ ^]^ or SP1.

As erythroid differentiation and α‐globin transcription proceed, the probability of interaction between the enhancers and promoters, as judged from tiled capture‐C and super‐resolution microscopy, appears to increase (Figure 2B).^[^ ^9^ ^]^ It may be that this is related to increased loop extrusion. In support of this, in recent experiments we have placed a CTCF‐bound insulator between the enhancers and the promoters and found that this significantly decreases α‐globin expression in an orientation‐dependent manner (Stolper R, Tsang F et al., in preparation). Of interest, the insulator causes a greater effect on α‐gene expression when orientated such that it would block cohesin translocating from the enhancer to the promoter. This suggests that loop extrusion is required for the interaction between the α‐globin enhancers and promoters and further work is underway to fully test this hypothesis. It remains possible that there is another as yet unknown mechanism by which insulators can alter enhancer‐promoter interactions or transcription in an orientation‐dependent manner. For example, it is possible that random diffusion of enhancers and promoters is limited by the 3D structure of the sub‐TAD which might be altered by the position and orientation of newly introduced CTCF insulators.

In summary, long‐range enhancer‐promoter communication could result from the combination of diffusion and loop extrusion bringing the enhancer and promoter in close proximity and bridging of TFs and co‐factors.

## WHAT IS THE NATURE OF THE ENHANCER‐PROMOTER INTERACTION?

It is generally agreed that enhancer‐driven transcription is associated with increased proximity between enhancers and their cognate promoters,^[^ ^62^, ^63^, ^64^, ^65^, ^66^ ^]^ although there are exceptions.^[^ ^67^, ^68^ ^]^ This is consistent with the concept that enhancers and activated promoters may be found in transcriptional hubs (originally referred to as transcription factories) in which there is a high concentration of the many factors and co‐factors required for transcription (e.g., TFs, PolII, Mediator and BRD4). Various models have been proposed describing the relationship between these proteins, chromatin, DNA and RNA including eRNAs. Although originally proposed as fixed nuclear sub‐structures, it has been shown that these hubs are more likely transient, non‐membrane bound nuclear compartments.^[^ ^69^ ^]^ The biophysical nature of these hubs is a matter of current debate and it has been proposed that the intrinsically disordered domains of proteins within the hubs may form liquid‐liquid phase separated condensates.^[^ ^70^, ^71^ ^]^ It has also been suggested that the transient nature of the hubs and interactions between proteins may also explain why transcription occurs in bursts often lasting minutes rather than occurring continuously.^[^ ^49^, ^72^, ^73^ ^]^

Using super‐resolution microscopy, we have observed the active α‐globin locus in structures consistent with loop‐extruded chromatin flanked by CTCF‐bound insulators (Figure 4B). This together with tiled capture C experiments shows that the enhancer and promoter come into close proximity only in erythroid cells.^[^ ^9^, ^50^ ^]^ Using tri‐C experiments, which capture interactions between regulatory elements in single cells, it appears that the five α‐globin enhancers work as a group rather than as individual elements.^[^ ^74^ ^]^ This observation is further supported by micro‐capture‐C (MCC), which shows at nucleotide‐resolution that the enhancers interact more frequently with each other than with the α‐globin promoters.^[^ ^60^ ^]^ In addition, this work suggests that the α‐globin enhancers not only interact with the globin genes but also with other flanking promoters in the same TAD, suggesting that the hub contains several transcriptionally active promoters. This is consistent with the model suggesting that an enhancer may activate more than one promoter at the same time.^[^ ^75^, ^76^, ^77^, ^78^ ^]^

The content, distribution, concentrations and roles of the very large number of proteins (histones, TFs, co‐factors, enzymes, etc.) thought to be contained within the transcriptional hub are sketchy. One important complex with 26 subunits that has been considered in detail is the Mediator complex and its associated protein Brd4. Subunits at the tail module of Mediator serve as a major interaction surface for a variety of sequence‐specific TFs located at the enhancer whilst the head module serves as a docking site of Pol II and general TFs^[^ ^79^, ^80^ ^]^ at the promoter. Depletion of Mediator globally diminishes the level of gene expression,^[^ ^81^ ^]^ but enhancer‐promoter interactions are largely preserved^[^ ^81^, ^82^ ^]^ or slightly reduced^[^ ^83^ ^]^ even after acute depletion of Mediator or PIC components implying that Mediator does not serve as a major structural bridge between enhancers and promoters.

In line with the hub hypothesis, the α‐genes, like many other genes, are transcribed in frequent transcriptional bursts each lasting ∼5 min. As in other systems, our findings suggest that the enhancers influence the frequency of transcriptional bursting.^[^ ^73^ ^]^ A key unanswered question is whether the transcriptional bursts result from direct physical interaction between the enhancer and promoter or more simply from transient assembly and dispersal of the transcriptionally favourable hub. Measuring dynamic changes in the distance between enhancers and promoters is challenging and at the limit of current imaging. Nevertheless, recent efforts to measure the distances between enhancers and their target promoters during active transcription suggest that, although in proximity, at the atomic scale they are separated by large distances, in the order of 200–300 nanometers (nm).^[^ ^49^, ^84^, ^85^ ^]^ Given the sizes of the proteins and molecular crowding within the hub, this separation may simply reflect the contact between multiprotein complexes binding to the enhancers and promoters.

MCC, a cell population assay, defines enhancer‐promoter interactions with base‐pair resolution,^[^ ^60^ ^]^ and this reveals the patterns of proteins interacting at the α‐globin enhancers and promoters. Whilst this suggests that there are atomic‐range interactions between these elements, it does not tell us how frequent they are or their relationship to transcription. Two elements separated by 200–300 nm might well contact each other by random diffusion and not be related to transcriptional activation. Models correlating contact and transcriptional activation may be further complicated by the requirement for multiple enhancer‐promoter contact events to activate transcription.^[^ ^63^ ^]^

## DO CLUSTERS OF ENHANCERS WORK AS A GROUP OR INDIVIDUALLY?

The visualisation of the extensive contacts between different cis‐regulatory elements in high resolution has led to the hypothesis that clusters of enhancers (SE) accentuate the impact of protein crowding for the formation of transcriptional hubs for gene regulation, and it brings to the fore the importance of understanding how these genomic elements function. The fundamental elements of the genome (enhancers, promoters and insulators) undoubtedly interact depending on their individual attributes and their distributions with respect to each other in the genome. The situation is made more complex because there is considerable overlap in the function of these elements which are very frequently initially classified by their locations with respect to transcriptional start sites and their epigenetic signatures. Most enhancers also act as promoters producing eRNAs, or meRNAs if located within the introns of a gene; the α‐gene enhancers are no exception.^[^ ^86^ ^]^ Some promoters can act as enhancers and sometimes may act as insulators, preventing an enhancer activating a more distal gene.^[^ ^87^, ^88^ ^]^

It has been estimated that each gene may be regulated by 40–50 enhancers: although this remains to be seen, it seems unlikely. Genome‐wide identification of enhancer elements based on their chromatin signatures does not correlate very well with the activity of such elements in classical enhancer assays. This suggests that many elements identified by current chromatin signatures are not enhancers and other regulatory elements such as tethering elements and recently described facilitators which have no intrinsic enhancer activity may share signatures that are currently indistinguishable from classical enhancers.^[^ ^42^, ^65^, ^66^ ^]^ The identification of as many as 11 different classes of multipartite clusters of enhancers including SEs and LCRs, has added to the complexity of enhancer biology.^[^ ^89^ ^]^ It is not clear to what extent these multipartite enhancers act simply as a group of conventional enhancers or whether they include other classes of regulatory elements. Furthermore, it is not clear if these multipartite enhancers cooperate additively or if they act together to be more than the sum of their parts.

**FIGURE 5:**
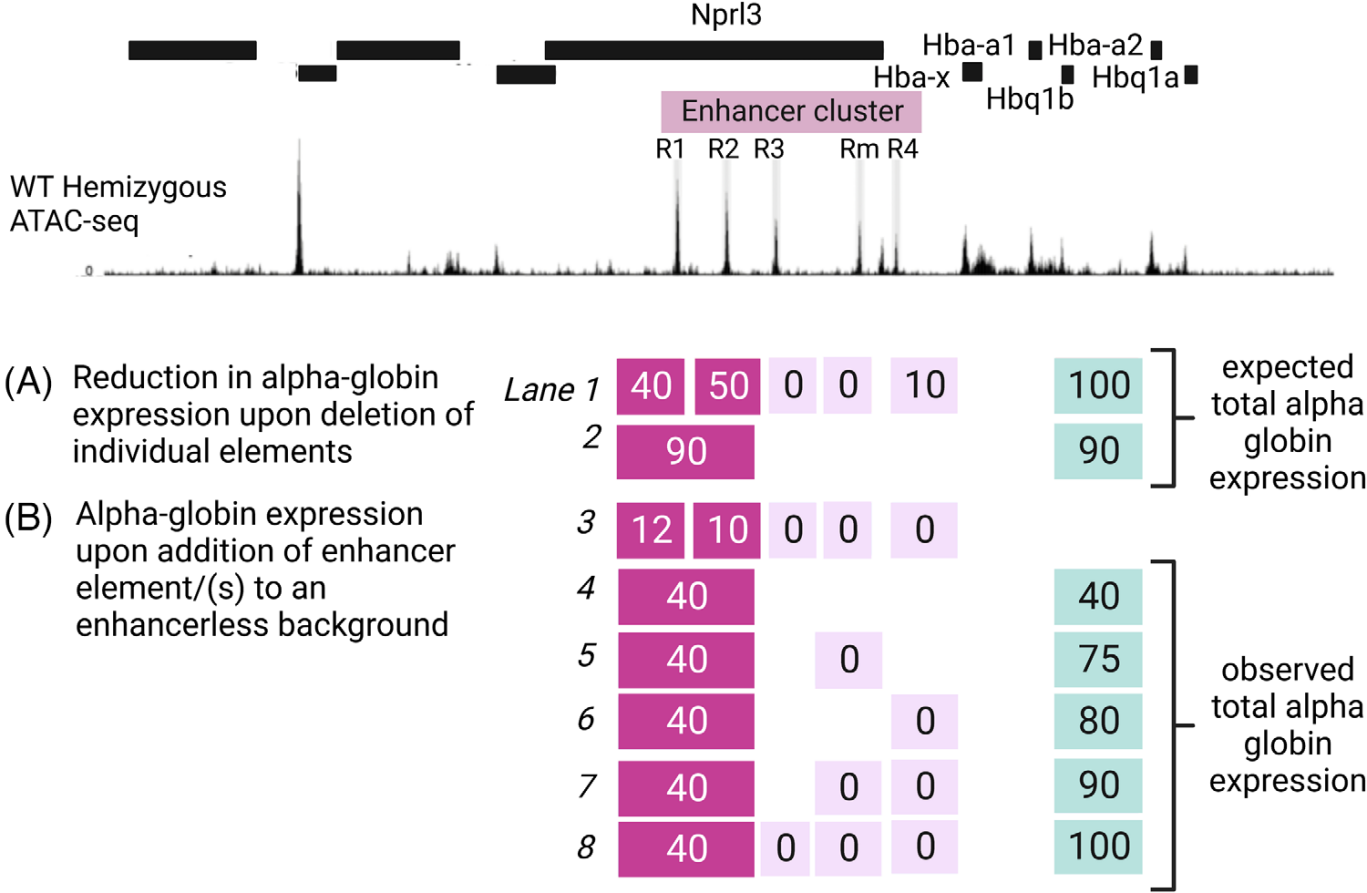
The cooperation of α‐globin super‐enhancer elements in driving gene expression. Top, UCSC Refseq gene annotation across the α‐globin locus and ATAC‐seq peaks highlighted in grey bars corresponding to the α‐globin SE constituent elements (R1, R2, R3, Rm, R4). (A) The reduction in α‐globin expression, as a percentage of a wildtype expression level, corresponding to SE elements upon deletion of a single element (lane 1, for example the deletion of R1 only causes a drop of 40% in expression whilst the single deletion of R3 causes no expression change) or a combination of elements (R1 and R2 deleted together whilst other elements remain intact, lane 2) as reported in ref.[14] (B) Contribution to α‐globin expression, presented as a percentage of a wildtype expression level, of single elements (lane 3, adding R1 only in the absence of all other elements contributed only 12% of expression whereas adding R3 caused no activation) or a combination of elements when added to an enhancer‐less α‐globin locus (lane 4: R1 + R2, lane 5: R1 + R2 + Rm, lane 6: R1 + R2 + R4, lane 7: R1 + R2 + Rm + R4, lane 8: R1 + R2 + R3 + Rm + R4) as reported in ref.[42] Note the discrepancy between the expected versus observed levels of expression based on deletion versus sequential addition of elements. R3, Rm and R4, inactive based on the deletion study (lane 1), prove crucial for the full activity of R1 and R2 elements (compare lanes 2 to 4 and 8).

We previously analysed potential synergy between the five α‐globin enhancers (R1, R2, R3, Rm and R4) by studying them in conventional enhancer assays and then by removing each enhancer individually and in informative but limited combinations from the endogenous cluster in mouse models. This showed that although all elements had the signature of an enhancer, most of the activity was encoded within R1 (40%) and R2 (50%) with R3, Rm and R4 having little or no activity when removed individually from the cluster. From these experiments, it appeared that the elements acted additively (Figure 5A).

To test this further, we recently rebuilt the cluster by generating an enhancerless allele and subsequently adding each element individually and in combination.^[^ ^42^ ^]^ This work showed that on their own, without the context of the other elements, R1 (10% transcription) and R2 (15% transcription) had much less activity than predicted from previous experiments (Figure 5A). Furthermore, sequential addition of the enhancer‐like elements, with no inherent enhancer activity, ultimately restored full function (Figure 5B). Importantly, the extent to which each element restored activity was dependent on its position relative to R1 and R2. We have called these elements facilitators and they share the known chromatin marks with other elements of the α‐globin enhancer cluster. It is worth noting that facilitators do not score in conventional enhancer assays and consequently are probably discarded as inactive enhancers. Their deletion and impact on gene expression in situ could be interpreted similarly to canonical enhancers, as necessary, redundant or inactive, obscuring their differing nature. Also, the fact that the facilitators only have activity in the presence of active enhancers further obscures assignment of their role within the cluster; if activators are deleted, target gene expression is totally abolished even if facilitators are intact. It is only by combining their various characteristics with extensive genetic dissection that their role in regulating optimal levels of target gene expression can be revealed. Identifying and analysing elements which serve this type of role in other multipartite enhancers will be important.

## DO CLUSTERS OF ENHANCERS WORK IN AN ORIENTATION DEPENDENT MANNER?

Individual enhancers, by definition, act in an orientation‐independent manner.^[^ ^90^, ^91^ ^]^ As the initial reports describing enhancers and their characteristics were plasmid‐based, the topic remains subject to debate. There are relatively few reports challenging the orientation of single enhancers in their natural chromatin context.^[^ ^92^, ^93^, ^94^ ^]^ We have inverted R2, the major enhancer element in the α‐globin enhancer cluster, both in human and mouse loci in erythroid cultures and showed no effect on the expression of α‐globin,^[^ ^92^, ^94^ ^]^ supporting a single‐enhancer orientation‐independent function. However, some reports highlight the promiscuity of enhancers and their ability to simultaneously control more than one gene including genes lying upstream and downstream of the enhancer.^[^ ^77^ ^]^ Also, in the context of chromatin, the organisation of the genome can act to constrain enhancer activity in one particular direction. For example, an enhancer located at the 5′ boundary of a TAD will preferentially interact with, and activate expression of, target genes located 3′ within the body of the TAD.^[^ ^95^, ^96^, ^97^ ^]^ The enhancer orientation‐independent function paradigm therefore should be revisited.

Equally, it is not known if clusters of enhancer‐like elements, working as a unit, harbour functional polarity. Of interest, in one set of experiments using large, randomly integrated transgenic inserts derived from bacterial artificial chromosomes, it was found that inversion of the β‐globin LCR (a well‐characterised cluster of enhancers) reduced expression of the linked β‐globin gene cluster.^[^ ^98^ ^]^ In addition, when the β‐globin LCR was inserted in either orientation within a group of housekeeping genes, although it activated genes both 5′ and 3′, the upregulated genes changed depending on the orientation of the LCR.^[^ ^99^ ^]^ Finally, when the complex enhancer cluster lying between the *Kcnj2* gene and the *Sox9* gene was inverted, there was a reciprocal change in expression of these two genes.^[^ ^100^ ^]^ Together, these observations suggest that, whereas single enhancers act in an orientation independent manner, some clusters of enhancers may act as a unit with an encoded bias to the direction in which they activate gene expression. However, it remains unclear whether such directionality is a general feature of SEs and what the underlying mechanisms might be.

We have recently analysed the direction of interaction and the effects on gene expression of the cluster of α‐globin enhancers.^[^ ^92^ ^]^ To examine any effect of the orientation of enhancer clusters on transcription, we used the mouse α‐globin locus as an experimental model. We have previously shown that the cluster of enhancers regulating α‐globin expression represents one of the most highly ranked SEs in erythroid cells^[^ ^14^ ^]^ . Although the α‐globin SE has a major influence on expression of the α‐globin genes lying 30 kb 3′, it has little activity on the genes within the ∼165 kb α‐globin TAD lying 12–35 kb upstream of R1.

**FIGURE 6:**
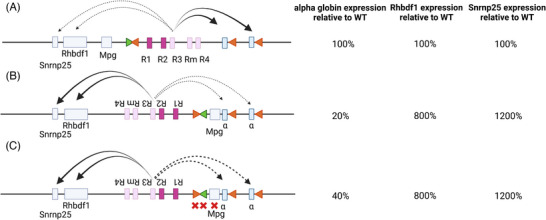
The α‐globin super‐enhancer functions in an orientation‐dependent manner. (A) The α‐globin SE in its native configuration preferentially interacts with the α‐globin genes in erythroid cells as schematically indicated and drives normal levels of expression, as presented in the table (indicated as 100%). The genes lying upstream of the α‐globin SE are also expressed, albeit at much lower levels than the α‐globin, in in vitro‐derived erythroid cells. (B) When inverted, the α‐globin SE interacts less with the α‐globin genes, which are now expressing at 20% of the wildtype levels in in vitro‐derived erythroid cells. The inverted SE interacts more with Rhbdf1 and Snrnp25, the genes lying 5′ of the native locus, in the direction of the inversion, upregulating both genes, as schematically shown and as reported in ref.[92] (C) Potential confounding factors, the CTCF boundary elements and the intervening MpG gene, were deleted. The phenotype persisted, demonstrating that the functional polarity is enhancer‐cluster driven.

We found that by inverting the entire α‐globin SE, with or without surrounding CTCF binding sites or intervening promoters, the predominant interactions from the SE change direction and while α‐globin expression is severely reduced, expression of the genes lying upstream, 5′ of the SE, is increased (Figure 6).^[^ ^92^ ^]^ Together, these findings show that clusters of enhancers (such as SEs), in contrast to individual enhancers, may interact and influence gene expression in an orientation‐dependent manner. This functional polarity is promoter agnostic and encoded within the cluster itself. At present it is not clear if the newly discovered facilitator elements whose activity depends on position play a role in determining the orientation of the activity of the cluster. We have described a functional hierarchy among the facilitators to be more dependent on each element's position with respect to the promoter rather than its sequence.^[^ ^42^ ^]^ This observation may shed light on possible mechanisms that contribute to the cluster's functional directionality. In the α‐globin SE inversion model, the position of R1 and R2 is almost unchanged, whereas the three facilitators are re‐oriented towards the upstream genes, suggesting that their re‐positioning may cause the associated changes in gene expression. We hypothesise that a unidirectional linear tracking mechanism, such as loop extrusion powered by cohesin,^[^ ^10^, ^56^ ^]^ may underlie the inherent functional orientation of a SE but this remains to be investigated.

## NATURAL VARIATION IN THE ENHANCERS AND HUMAN DISEASE

Over the past 20 years, it has emerged that most genetic variation associated with common traits (e.g., height and weight) and risk of complex genetic disease (e.g., diabetes, hypertension, asthma) occurs in the non‐coding genome and particularly in enhancer elements.^[^ ^101^, ^102^, ^103^ ^]^ It is becoming increasingly clear that the genetic, structural and/or epigenetic disruption of enhancers represent major causative factors in many human diseases referred to as enhanceropathies, ranging from rare congenital disorders to common diseases associated with ageing and lifestyle (e.g., cancer, diabetes). In most cases, the target genes remain unknown and in those where the genes have been identified, the mechanisms by which these variants change gene expression in most cases are unknown.^[^ ^104^, ^105^, ^106^ ^]^ By contrast, mutations causing well defined monogenic diseases are most often found in the coding sequences of well‐defined genes; these include single nucleotide variants and insertion/deletions.^[^ ^107^ ^]^ In monogenic diseases, such mutations, perturbing binding of specific TFs, are rarely found in distal enhancers. By contrast rare deletions, duplications, translocations or inversions of enhancers have been reported (reviewed ^[^ ^108^, ^109^, ^110^ ^]^).

**FIGURE 7:**
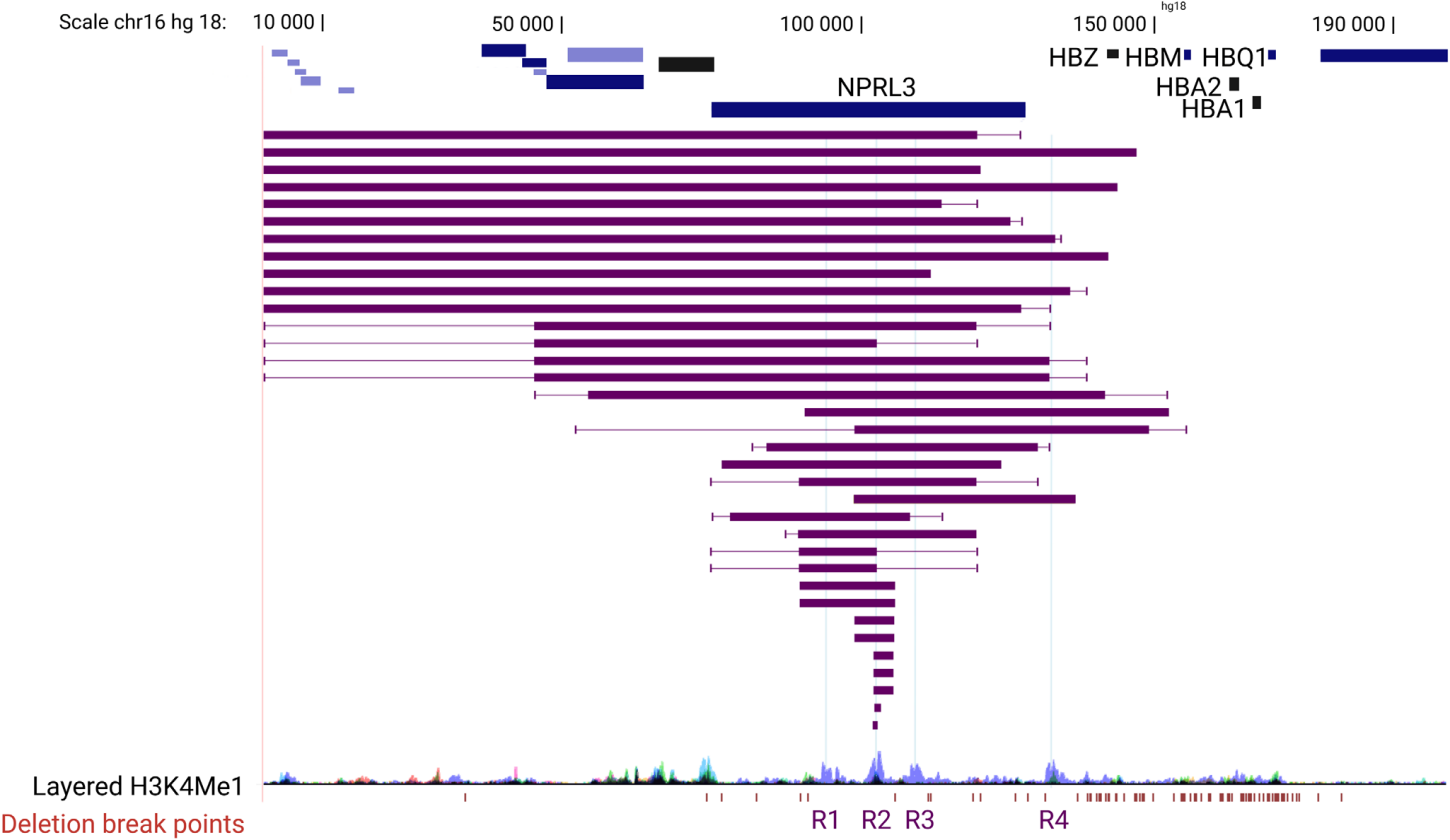
Deletions encompassing the α‐globin enhancer cluster which give rise to α‐thalassemia.

Consistent with these general observations, common natural variation of the human α‐globin cluster causing α‐thalassaemia is almost always due to deletions or nucleotide variants in the coding sequence. Nevertheless, deletions and duplications of the α‐globin enhancer elements have been seen in sporadic families with α‐thalassaemia (Figure 7), often from geographical regions where α‐thalassaemia is otherwise rare. These have arisen by illegitimate recombination, telomeric truncation and translocation of the enhancers. These rare families provided some of the first examples of human enhanceropathies caused by deletion of enhancers and first pointed to the existence of distal regulatory elements controlling α‐globin gene expression. Alpha‐globin enhanceropathies remove between one and all four of the human α‐globin enhancer elements, and of interest, all of the deletions characterised to date include R2. This is a classical enhancer that, in humans, contributes 90% of enhancer activity of the cluster of four α‐globin enhancers. One patient homozygous for a deletion of R2 had a moderately severe anaemia showing that the remaining enhancers can drive sufficient α‐globin gene expression to sustain a relatively normal life, supporting the idea that R1 together with R3 and R4 can act as ‘shadow’ enhancers, seemingly redundant enhancers but shown to add robustness to tissue‐ and developmental‐specific gene expression.^[^ ^94^, ^111^ ^]^ Despite careful analysis of ∼50 individuals with the phenotype of α‐thalassaemia and yet no associated deletions or insertions, we have never observed a deleterious single nucleotide polymorphism in the R2 enhancer. Although such polymorphisms may exist, it is likely that they do not cause sufficient change in α‐globin expression to cause a recognisable change in phenotype.

## CONCLUSIONS AND FUTURE QUESTIONS

The key challenges of understanding how enhancers activate gene expression and their role in disease have been well summarised. Clearly, enhancer(s) must be securely assigned to their specific targets. Importantly we need a consistent definition of an enhancer based on its activity within its normal chromosomal context rather than in transient assays, randomly integrated transgenes, or on the basis of a chromatin signature. The correlation between enhancer signatures and activities is poor. Most enhancers work in specific cellular contexts and must be tested in these contexts. Analysing enhancer‐gene pairs that alter cell fate results in difficulties in interpreting changes in gene expression since the entire transcriptional and epigenetic programme will have changed, potentially producing secondary effects on expression of the gene in question.

The α‐globin model discussed here fulfils all of these criteria and is allowing us to ask the fundamental general questions about enhancer‐promoter biology. First, to fully characterise the individual elements in the cluster of α‐globin enhancers: they are not all classical enhancers. Second, to ask about enhancer‐promoter compatibility: the α‐globin enhancers preferentially activate the α‐globin promoters rather than other promoters that lie closer to them in linear proximity. Third, to address how distal enhancers come into proximity with their cognate promoters: it appears that loop extrusion plays some role in this but may not provide a full explanation. The recent discovery of a role for the orientation of the α‐globin enhancers in promoter choice is of great interest, particularly in the context of a linear tracking model of cohesin‐mediated enhancer‐promoter proximity. Finally, the nature of the transcriptional hub and its relationship to transcriptional bursting needs further examination: we need to understand the anatomy of a hub at the nanoscale and the dynamics of DNA, RNA and proteins within such structures. Although there will be variations on the mechanisms elucidated by studying the α‐globin locus, the history of our understanding of mammalian gene regulation from the principles established at the globin loci suggest that the fundamental underlying mechanisms may be very similar.

The final arbiter of whether or not we fully understand how enhancers activate transcription from their cognate promoters will be to build an accurately regulated locus from scratch in a neutral region of the genome using synthetic biology. This approach is now possible and underway.

## AUTHOR CONTRIBUTION

Mira Kassouf and Doug Higgs: Writing‐original draft. Mira Kassouf, Doug Higgs, Seren Ford and Joseph Blayney: Writing‐review, editing and figures. Doug Higgs: Funding acquisition.

## CONFLICT OF INTEREST STATEMENT

The authors declare no conflicts of interest.

## Data Availability

Data sharing is not applicable to this article as no datasets were generated or analysed specifically for this review.

## References

[1] The ENCODE Project Consortium. Abascal, F. , Acosta, R. , Addleman, N. J. , Adrian, J. , Afzal, V. , Ai, R. , Aken, B. , Akiyama, J. A. , Jammal, O. A. , Amrhein, H. , Anderson, S. M. , Andrews, G. R. , Antoshechkin, I. , Ardlie, K. G. , Armstrong, J. , Astley, M. , Banerjee, B. , Barkal, A. A. , … Weng, Z. (2020). Expanded encyclopaedias of DNA elements in the human and mouse genomes. Nature, 583(7818), 699–710. 10.1038/s41586-020-2493-4 32728249 PMC7410828

[2] Kim, T. H. , Abdullaev, Z. K. , Smith, A. D. , Ching, K. A. , Loukinov, D. I. , Green, R. D. , Zhang, M. Q. , Lobanenkov, V. V. , & Ren, B. (2007). Analysis of the vertebrate insulator protein CTCF‐binding sites in the human genome. Cell, 128(6), 1231–1245. 10.1016/j.cell.2006.12.048 17382889 PMC2572726

[3] Xie, X. , Mikkelsen, T. S. , Gnirke, A. , Lindblad‐Toh, K. , Kellis, M. , & Lander, E. S. (2007). Systematic discovery of regulatory motifs in conserved regions of the human genome, including thousands of CTCF insulator sites. Proceedings of the National Academy of Sciences, 104(17), 7145–7150. 10.1073/pnas.0701811104 PMC1852749 17442748

[4] Sender, R. , & Milo, R. (2021). The distribution of cellular turnover in the human body. Nature Medicine, 27(1), 45–48. 10.1038/s41591-020-01182-9 33432173

[5] Palis, J. (2014). Primitive and definitive erythropoiesis in mammals. Frontiers in Physiology, 5, 3. 10.3389/fphys.2014.00003 24478716 PMC3904103

[6] Ludwig, L. S. , Lareau, C. A. , Bao, E. L. , Nandakumar, S. K. , Muus, C. , Ulirsch, J. C. , Chowdhary, K. , Buenrostro, J. D. , Mohandas, N. , An, X. , Aryee, M. J. , Regev, A. , & Sankaran, V. G. (2019). Transcriptional states and chromatin accessibility underlying human erythropoiesis. Cell Reports, 27(11), 3228–3240.e7. 10.1016/j.celrep.2019.05.046 31189107 PMC6579117

[7] Pop, R. , Shearstone, J. R. , Shen, Q. , Liu, Y. , Hallstrom, K. , Koulnis, M. , Gribnau, J. , & Socolovsky, M. (2010). A key commitment step in erythropoiesis is synchronized with the cell cycle clock through mutual inhibition between PU.1 and S‐phase progression. PLoS Biology, 8(9), e1000484. 10.1371/journal.pbio.1000484 20877475 PMC2943437

[8] Harteveld, C. L. , & Higgs, D. R. (2010). α‐thalassaemia. Orphanet Journal of Rare Diseases, 5(1), 13. 10.1186/1750-1172-5-13 20507641 PMC2887799

[9] Oudelaar, A. M. , Beagrie, R. A. , Gosden, M. , de Ornellas, S. , Georgiades, E. , Kerry, J. , Hidalgo, D. , Carrelha, J. , Shivalingam, A. , El‐Sagheer, A. H. , Telenius, J. M. , Brown, T. , Buckle, V. J. , Socolovsky, M. , Higgs, D. R. , & Hughes, J. R. (2020). Dynamics of the 4D genome during in vivo lineage specification and differentiation. Nature Communications, 11(1), 2722. 10.1038/s41467-020-16598-7 PMC7264236 32483172

[10] Fudenberg, G. , Imakaev, M. , Lu, C. , Goloborodko, A. , Abdennur, N. , & Mirny, L. A. (2016). Formation of chromosomal domains by loop extrusion. Cell Reports, 15(9), 2038–2049. 10.1016/j.celrep.2016.04.085 27210764 PMC4889513

[11] Dixon, J. R. , Selvaraj, S. , Yue, F. , Kim, A. , Li, Y. , Shen, Y. , Hu, M. , Liu, J. S. , & Ren, B. (2012). Topological domains in mammalian genomes identified by analysis of chromatin interactions. Nature, 485(7398), 376–380. 10.1038/nature11082 22495300 PMC3356448

[12] Rao, S. S. P. , Huntley, M. H. , Durand, N. C. , Stamenova, E. K. , Bochkov, I. D. , Robinson, J. T. , Sanborn, A. L. , Machol, I. , Omer, A. D. , Lander, E. S. , & Aiden, E. L. (2014). A 3D map of the human genome at kilobase resolution reveals principles of chromatin looping. Cell, 159(7), 1665–1680. 10.1016/j.cell.2014.11.021 25497547 PMC5635824

[13] Whyte, W. , Orlando, D. , Hnisz, D. , Abraham, B. , Lin, C. , Kagey, M. , Rahl, P. , Lee, T. , & Young, R. (2013). Master transcription factors and mediator establish super‐enhancers at key cell identity genes. Cell, 153(2), 307–319. 10.1016/j.cell.2013.03.035 23582322 PMC3653129

[14] Hay, D. , Hughes, J. R. , Babbs, C. , Davies, J. O. J. , Graham, B. J. , Hanssen, L. L. P. , Kassouf, M. T. , Oudelaar, A. M. , Sharpe, J. A. , Suciu, M. C. , Telenius, J. , Williams, R. , Rode, C. , Li, P.‐S. , Pennacchio, L. A. , Sloane‐Stanley, J. A. , Ayyub, H. , Butler, S. , Sauka‐Spengler, T. , … Higgs, D. R. (2016). Genetic dissection of the α‐globin super‐enhancer in vivo. Nature Genetics, 48(8), 895–903. 10.1038/ng.3605 27376235 PMC5058437

[15] Hanssen, L. L. P. , Kassouf, M. T. , Oudelaar, A. M. , Biggs, D. , Preece, C. , Downes, D. J. , Gosden, M. , Sharpe, J. A. , Sloane‐Stanley, J. A. , Hughes, J. R. , Davies, B. , & Higgs, D. R. (2017). Tissue‐specific CTCF – cohesin‐mediated chromatin architecture delimits enhancer interactions and function in vivo. Nature Cell Biology, 19(8), 952–961. 10.1038/ncb3573 28737770 PMC5540176

[16] Hughes, J. R. , Cheng, J.‐F. , Ventress, N. , Prabhakar, S. , Clark, K. , Anguita, E. , Gobbi, M. D. , de Jong, P. , Rubin, E. , & Higgs, D. R. (2005). Annotation of cis‐regulatory elements by identification, subclassification, and functional assessment of multispecies conserved sequences. Proceedings of the National Academy of Sciences, 102(28), 9830–9835. 10.1073/pnas.0503401102 PMC1174996 15998734

[17] Tusi, B. K. , Wolock, S. L. , Weinreb, C. , Hwang, Y. , Hidalgo, D. , Zilionis, R. , Waisman, A. , Huh, J. R. , Klein, A. M. , & Socolovsky, M. (2018). Population snapshots predict early haematopoietic and erythroid hierarchies. Nature, 555(7694), 54–60. 10.1038/nature25741 29466336 PMC5899604

[18] Garrick, D. , Gobbi, M. D. , Samara, V. , Rugless, M. , Holland, M. , Ayyub, H. , Lower, K. , Sloane‐Stanley, J. , Gray, N. , Koch, C. , Dunham, I. , & Higgs, D. R. (2008). The role of the polycomb complex in silencing α‐globin gene expression in nonerythroid cells. Blood, 112(9), 3889–3899. 10.1182/blood-2008-06-161901 18689541 PMC2572806

[19] Vernimmen, D. , Lynch, M. D. , Gobbi, M. D. , Garrick, D. , Sharpe, J. A. , Sloane‐Stanley, J. A. , Smith, A. J. H. , & Higgs, D. R. (2011). Polycomb eviction as a new distant enhancer function. Genes & Development, 25(15), 1583–1588. 10.1101/gad.16985411 21828268 PMC3182025

[20] Herz, H.‐M. , Mohan, M. , Garruss, A. S. , Liang, K. , Takahashi, Y. , Mickey, K. , Voets, O. , Verrijzer, C. P. , & Shilatifard, A. (2012). Enhancer‐associated H3K4 monomethylation by trithorax‐related, the ∖emphDrosophila homolog of mammalian Mll3/Mll4. Genes & Development, 26(23), 2604–2620. 10.1101/gad.201327.112 23166019 PMC3521626

[21] Hu, D. , Gao, X. , Morgan, M. A. , Herz, H.‐M. , Smith, E. R. , & Shilatifard, A. (2013). The MLL3/MLL4 branches of the COMPASS family function as major histone H3K4 monomethylases at enhancers. Molecular and Cellular Biology, 33(23), 4745–4754. 10.1128/mcb.01181-13 24081332 PMC3838007

[22] Rickels, R. , Hu, D. , Collings, C. K. , Woodfin, A. R. , Piunti, A. , Mohan, M. , Herz, H.‐M. , Kvon, E. , & Shilatifard, A. (2016). An evolutionary conserved epigenetic mark of polycomb response elements implemented by Trx/MLL/COMPASS. Molecular Cell, 63(2), 318–328. 10.1016/j.molcel.2016.06.018 27447986 PMC4996622

[23] Zaret, K. S. , & Carroll, J. S. (2011). Pioneer transcription factors: Establishing competence for gene expression. Genes & Development, 25(21), 2227–2241. 10.1101/gad.176826.111 22056668 PMC3219227

[24] Ohneda, K. , Ohmori, S. , Ishijima, Y. , Nakano, M. , & Yamamoto, M. (2009). Characterization of a functional ZBP‐89 binding site that mediates gata1 gene expression during hematopoietic development. Journal of Biological Chemistry, 284(44), 30187–30199. 10.1074/jbc.m109.026948 19723625 PMC2781574

[25] Cantor, A. B. , & Orkin, S. H. (2002). Transcriptional regulation of erythropoiesis: An affair involving multiple partners. Oncogene, 21(21), 3368–3376. 10.1038/sj.onc.1205326 12032775

[26] Shilatifard, A. (2012). The COMPASS family of histone H3K4 methylases: Mechanisms of regulation in development and disease pathogenesis. Annual Review of Biochemistry, 81(1), 65–95. 10.1146/annurev-biochem-051710-134100 PMC4010150 22663077

[27] Ashe, H. L. , Monks, J. , Wijgerde, M. , Fraser, P. , & Proudfoot, N. J. (1997). Intergenic transcription and transinduction of the human β‐globin locus. Genes & Development, 11(19), 2494–2509. 10.1101/gad.11.19.2494 9334315 PMC316561

[28] Mousavi, K. , Zare, H. , Dell'Orso, S. , Grontved, L. , Gutierrez‐Cruz, G. , Derfoul, A. , Hager, G. , & Sartorelli, V. (2013). eRNAs promote transcription by establishing chromatin accessibility at defined genomic loci. Molecular Cell, 51(5), 606–617. 10.1016/j.molcel.2013.07.022 23993744 PMC3786356

[29] Kim, Y. , Lee, S. , Yun, J. , & Kim, A. (2015). Chromatin looping and eRNA transcription precede the transcriptional activation of gene in the β‐globin locus. Bioscience Reports, 35(2), e00179. 10.1042/bsr20140126 25588787 PMC4370096

[30] Li, W. , Notani, D. , Ma, Q. , Tanasa, B. , Nunez, E. , Chen, A. Y. , Merkurjev, D. , Zhang, J. , Ohgi, K. , Song, X. , Oh, S. , Kim, H.‐S. , Glass, C. K. , & Rosenfeld, M. G. (2013). Functional roles of enhancer RNAs for oestrogen‐dependent transcriptional activation. Nature, 498(7455), 516–520. 10.1038/nature12210 23728302 PMC3718886

[31] Arnold, P. R. , Wells, A. D. , & Li, X. C. (2020). Diversity and emerging roles of enhancer RNA in regulation of gene expression and cell fate. Frontiers in Cell and Developmental Biology, 7, 377. 10.3389/fcell.2019.00377 31993419 PMC6971116

[32] Heintzman, N. D. , Stuart, R. K. , Hon, G. , Fu, Y. , Ching, C. W. , Hawkins, R. D. , Barrera, L. O. , Calcar, S. V. , Qu, C. , Ching, K. A. , Wang, W. , Weng, Z. , Green, R. D. , Crawford, G. E. , & Ren, B. (2007). Distinct and predictive chromatin signatures of transcriptional promoters and enhancers in the human genome. Nature Genetics, 39(3), 311–318. 10.1038/ng1966 17277777

[33] Roth, J.‐F. (2003). Differential role of P300 and CBP acetyltransferase during myogenesis: P300 acts upstream of MyoD and Myf5. The EMBO Journal, 22(19), 5186–5196. 10.1093/emboj/cdg473 14517256 PMC204457

[34] Kharchenko, P. V. , Alekseyenko, A. A. , Schwartz, Y. B. , Minoda, A. , Riddle, N. C. , Ernst, J. , Sabo, P. J. , Larschan, E. , Gorchakov, A. A. , Gu, T. , Linder‐Basso, D. , Plachetka, A. , Shanower, G. , Tolstorukov, M. Y. , Luquette, L. J. , Xi, R. , Jung, Y. L. , Park, R. W. , Bishop, E. P. , … Park, P. J. (2011). Comprehensive analysis of the chromatin landscape in drosophila melanogaster. Nature, 471(7339), 480–485. 10.1038/nature09725 21179089 PMC3109908

[35] Moriguchi, T. , & Yamamoto, M. (2014). A regulatory network governing Gata1 and Gata2 gene transcription orchestrates erythroid lineage differentiation. International Journal of Hematology, 100(5), 417–424. 10.1007/s12185-014-1568-0 24638828

[36] Gillespie, M. A. , Palii, C. G. , Sanchez‐Taltavull, D. , Shannon, P. , Longabaugh, W. J. R. , Downes, D. J. , Sivaraman, K. , Espinoza, H. M. , Hughes, J. R. , Price, N. D. , Perkins, T. J. , Ranish, J. A. , & Brand, M. (2020). Absolute quantification of transcription factors reveals principles of gene regulation in erythropoiesis. Molecular Cell, 78(5), 960–974.e11. 10.1016/j.molcel.2020.03.031 32330456 PMC7344268

[37] Wadman, I. A. (1997). The LIM‐only protein Lmo2 is a bridging molecule assembling an erythroid, DNA‐binding complex which includes the TAL1, E47, GATA‐1 and Ldb1/NLI proteins. The EMBO Journal, 16(11), 3145–3157. 10.1093/emboj/16.11.3145 9214632 PMC1169933

[38] Osada, H. , Grutz, G. , Axelson, H. , Forster, A. , & Rabbitts, T. H. (1995). Association of erythroid transcription factors: Complexes involving the LIM protein RBTN2 and the zinc‐finger protein GATA1. Proceedings of the National Academy of Sciences, 92(21), 9585–9589. 10.1073/pnas.92.21.9585 PMC40846 7568177

[39] Vernimmen, D. , Gobbi, M. D. , Sloane‐Stanley, J. A. , Wood, W. G. , & Higgs, D. R. (2007). Long‐range chromosomal interactions regulate the timing of the transition between poised and active gene expression. The EMBO Journal, 26(8), 2041–2051. 10.1038/sj.emboj.7601654 17380126 PMC1852780

[40] Funnell, A. P. , Vernimmen, D. , Lim, W. F. , Mak, K. S. , Wienert, B. , Martyn, G. E. , Artuz, C. M. , Burdach, J. , Quinlan, K. G. , Higgs, D. R. , Whitelaw, E. , Pearson, R. C. , & Crossley, M. (2014). Differential regulation of the α‐globin locus by krüppel‐like factor 3 in erythroid and non‐erythroid cells. BMC Molecular Biology, 15(1), 8. 10.1186/1471-2199-15-8 24885809 PMC4033687

[41] Larke, M. S. C. , Schwessinger, R. , Nojima, T. , Telenius, J. , Beagrie, R. A. , Downes, D. J. , Oudelaar, A. M. , Truch, J. , Graham, B. , Bender, M. A. , Proudfoot, N. J. , Higgs, D. R. , & Hughes, J. R. (2021). Enhancers predominantly regulate gene expression during differentiation via transcription initiation. Molecular Cell, 81(5), 983–997.e7. 10.1016/j.molcel.2021.01.002 33539786 PMC7612206

[42] Blayney, J. , Francis, H. , Camellato, B. , Mitchell, L. , Stolper, R. , Boeke, J. , Higgs, D. , & Kassouf, M. (2022). Super‐enhancers require a combination of classical enhancers and novel facilitator elements to drive high levels of gene expression. BioRxiv, 10.1101/2022.06.20.496856

[43] Hou, C. , Dale, R. , & Dean, A. (2010). Cell type specificity of chromatin organization mediated by CTCF and cohesin. Proceedings of the National Academy of Sciences, 107(8), 3651–3656. 10.1073/pnas.0912087107 PMC2840441 20133600

[44] Lin, C. , Garruss, A. , Luo, Z. , Guo, F. , & Shilatifard, A. (2013). The RNA Pol II elongation factor Ell3 marks enhancers in ES cells and primes future gene activation. Cell, 152(1–2), 144–156. 10.1016/j.cell.2012.12.015 23273992 PMC3556173

[45] Schoenfelder, S. , & Fraser, P. (2019). Long‐range enhancer – promoter contacts in gene expression control. Nature Reviews Genetics, 20(8), 437–455. 10.1038/s41576-019-0128-0 31086298

[46] Flyamer, I. M. , Gassler, J. , Imakaev, M. , Brandão, H. B. , Ulianov, S. V. , Abdennur, N. , Razin, S. V. , Mirny, L. A. , & Tachibana‐Konwalski, K. (2017). Single‐nucleus Hi‐C reveals unique chromatin reorganization at oocyte‐to‐zygote transition. Nature, 544(7648), 110–114. 10.1038/nature21711 28355183 PMC5639698

[47] Gabriele, M. , Brandão, H. B. , Grosse‐Holz, S. , Jha, A. , Dailey, G. M. , Cattoglio, C. , Hsieh, T.‐H. S. , Mirny, L. , Zechner, C. , & Hansen, A. S. (2022). Dynamics of CTCF‐ and cohesin‐mediated chromatin looping revealed by live‐cell imaging. Science, 376(6592), 496–501. 10.1126/science.abn6583 35420890 PMC9069445

[48] Mach, P. , Kos, P. I. , Zhan, Y. , Cramard, J. , Gaudin, S. , Tünnermann, J. , Marchi, E. , Eglinger, J. , Zuin, J. , Kryzhanovska, M. , Smallwood, S. , Gelman, L. , Roth, G. , Nora, E. P. , Tiana, G. , & Giorgetti, L. (2022). Live‐cell imaging and physical modeling reveal control of chromosome folding dynamics by cohesin and CTCF. BioRxiv, 10.1101/2022.03.03.482826 PMC9729113 36471076

[49] Li, J. , Dong, A. , Saydaminova, K. , Chang, H. , Wang, G. , Ochiai, H. , Yamamoto, T. , & Pertsinidis, A. (2019). Single‐molecule nanoscopy elucidates RNA polymerase II transcription at single genes in live cells. Cell, 178(2), 491–506.e28. 10.1016/j.cell.2019.05.029 31155237 PMC6675578

[50] Brown, J. M. , Roberts, N. A. , Graham, B. , Waithe, D. , Lagerholm, C. , Telenius, J. M. , Ornellas, S. D. , Oudelaar, A. M. , Scott, C. , Szczerbal, I. , Babbs, C. , Kassouf, M. T. , Hughes, J. R. , Higgs, D. R. , & Buckle, V. J. (2018). A tissue‐specific self‐interacting chromatin domain forms independently of enhancer‐promoter interactions. Nature Communications, 9(1), 3849. 10.1038/s41467-018-06248-4 PMC6155075 30242161

[51] Chen, L.‐F. , Lee, J. , & Boettiger, A. (2023). Recent progress and challenges in single‐cell imaging of enhancer – promoter interaction. Current Opinion in Genetics & Development, 79, 102023. 10.1016/j.gde.2023.102023 36854248

[52] Hafner, A. , & Boettiger, A. (2023). The spatial organization of transcriptional control. Nature Reviews Genetics, 24(1), 53–68. 10.1038/s41576-022-00526-0 36104547

[53] Karpinska, M. A. , & Oudelaar, A. M. (2023). The role of loop extrusion in enhancer‐mediated gene activation. Current Opinion in Genetics & Development, 79, 102022. 10.1016/j.gde.2023.102022 36842325

[54] Galouzis, C. C. , & Furlong, E. E. M. (2022). Regulating specificity in enhancer – promoter communication. Current Opinion in Cell Biology, 75, 102065. 10.1016/j.ceb.2022.01.010 35240372

[55] Nuebler, J. , Fudenberg, G. , Imakaev, M. , Abdennur, N. , & Mirny, L. A. (2018). Chromatin organization by an interplay of loop extrusion and compartmental segregation. Proceedings of the National Academy of Sciences, 115(29), E6697–E6706. 10.1073/pnas.1717730115 PMC6055145 29967174

[56] Banigan, E. J. , & Mirny, L. A. (2020). Loop extrusion: Theory meets single‐molecule experiments. Current Opinion in Cell Biology, 64, 124–138. 10.1016/j.ceb.2020.04.011 32534241

[57] Lim, B. , & Levine, M. S. (2021). Enhancer‐promoter communication: Hubs or loops? Current Opinion in Genetics & Development, 67, 5–9. 10.1016/j.gde.2020.10.001 33202367 PMC8653970

[58] Furlong, E. E. M. , & Levine, M. (2018). Developmental enhancers and chromosome topology. Science, 361(6409), 1341–1345. 10.1126/science.aau0320 30262496 PMC6986801

[59] Ciosk, R. , Shirayama, M. , Shevchenko, A. , Tanaka, T. , Toth, A. , Shevchenko, A. , & Nasmyth, K. (2000). Cohesin's binding to chromosomes depends on a separate complex consisting of Scc2 and Scc4 proteins. Molecular Cell, 5(2), 243–254. 10.1016/s1097-2765(00)80420-7 10882066

[60] Hua, P. , Badat, M. , Hanssen, L. L. P. , Hentges, L. D. , Crump, N. , Downes, D. J. , Jeziorska, D. M. , Oudelaar, A. M. , Schwessinger, R. , Taylor, S. , Milne, T. A. , Hughes, J. R. , Higgs, D. R. , & Davies, J. O. J. (2021). Defining genome architecture at base‐pair resolution. Nature, 595(7865), 125–129. 10.1038/s41586-021-03639-4 34108683

[61] Deng, W. , Lee, J. , Wang, H. , Miller, J. , Reik, A. , Gregory, P. D. , Dean, A. , & Blobel, G. A. (2012). Controlling long‐range genomic interactions at a native locus by targeted tethering of a looping factor. Cell, 149(6), 1233–1244. 10.1016/j.cell.2012.03.051 22682246 PMC3372860

[62] Chen, H. , Levo, M. , Barinov, L. , Fujioka, M. , Jaynes, J. B. , & Gregor, T. (2018). Dynamic interplay between enhancer – promoter topology and gene activity. Nature Genetics, 50(9), 1296–1303. 10.1038/s41588-018-0175-z 30038397 PMC6119122

[63] Zuin, J. , Roth, G. , Zhan, Y. , Cramard, J. , Redolfi, J. , Piskadlo, E. , Mach, P. , Kryzhanovska, M. , Tihanyi, G. , Kohler, H. , Eder, M. , Leemans, C. , van Steensel, B. , Meister, P. , Smallwood, S. , & Giorgetti, L. (2022). Nonlinear control of transcription through enhancer – promoter interactions. Nature, 604(7906), 571–577. 10.1038/s41586-022-04570-y 35418676 PMC9021019

[64] Rinzema, N. J. , Sofiadis, K. , Tjalsma, S. J. D. , Verstegen, M. J. A. M. , Oz, Y. , Valdes‐Quezada, C. , Felder, A.‐K. , Filipovska, T. , van der Elst, S. , de Andrade dos Ramos, Z. , Han, R. , Krijger, P. H. L. , & de Laat, W. (2022). Building regulatory landscapes reveals that an enhancer can recruit cohesin to create contact domains, engage CTCF sites and activate distant genes. Nature Structural & Molecular Biology, 29(6), 563–574. 10.1038/s41594-022-00787-7 PMC9205769 35710842

[65] Batut, P. J. , Bing, X. Y. , Sisco, Z. , Raimundo, J. , Levo, M. , & Levine, M. S. (2022). Genome organization controls transcriptional dynamics during development. Science, 375(6580), 566–570. 10.1126/science.abi7178 35113722 PMC10368186

[66] Calhoun, V. C. , Stathopoulos, A. , & Levine, M. (2002). Promoter‐proximal tethering elements regulate enhancer‐promoter specificity in the Drosophila Antennapedia complex. Proceedings of the National Academy of Sciences, 99(14), 9243–9247. 10.1073/pnas.142291299 PMC123125 12093913

[67] Benabdallah, N. S. , Williamson, I. , Illingworth, R. S. , Kane, L. , Boyle, S. , Sengupta, D. , Grimes, G. R. , Therizols, P. , & Bickmore, W. A. (2019). Decreased enhancer‐promoter proximity accompanying enhancer activation. Molecular Cell, 76(3), 473–484.e7. 10.1016/j.molcel.2019.07.038 31494034 PMC6838673

[68] Alexander, J. M. , Guan, J. , Li, B. , Maliskova, L. , Song, M. , Shen, Y. , Huang, B. , Lomvardas, S. , & Weiner, O. D. (2019). Live‐cell imaging reveals enhancer‐dependent Sox2 transcription in the absence of enhancer proximity. ELife, 8, e41769. 10.7554/elife.41769 31124784 PMC6534382

[69] Cisse, I. I. , Izeddin, I. , Causse, S. Z. , Boudarene, L. , Senecal, A. , Muresan, L. , Dugast‐Darzacq, C. , Hajj, B. , Dahan, M. , & Darzacq, X. (2013). Real‐time dynamics of RNA polymerase II clustering in live human cells. Science, 341(6146), 664–667. 10.1126/science.1239053 23828889

[70] Boija, A. , Klein, I. A. , Sabari, B. R. , Dall'Agnese, A. , Coffey, E. L. , Zamudio, A. V. , Li, C. H. , Shrinivas, K. , Manteiga, J. C. , Hannett, N. M. , Abraham, B. J. , Afeyan, L. K. , Guo, Y. E. , Rimel, J. K. , Fant, C. B. , Schuijers, J. , Lee, T. I. , Taatjes, D. J. , & Young, R. A. (2018). Transcription factors activate genes through the phase‐separation capacity of their activation domains. Cell, 175(7), 1842–1855.e16. 10.1016/j.cell.2018.10.042 30449618 PMC6295254

[71] Hnisz, D. , Shrinivas, K. , Young, R. A. , Chakraborty, A. K. , & Sharp, P. A. (2017). A phase separation model for transcriptional control. Cell, 169(1), 13–23. 10.1016/j.cell.2017.02.007 28340338 PMC5432200

[72] Tunnacliffe, E. , & Chubb, J. R. (2020). What is a transcriptional burst? Trends in Genetics, 36(4), 288–297. 10.1016/j.tig.2020.01.003 32035656

[73] Jeziorska, D. M. , Tunnacliffe, E. A. J. , Brown, J. M. , Ayyub, H. , Sloane‐Stanley, J. , Sharpe, J. A. , Lagerholm, B. C. , Babbs, C. , Smith, A. J. H. , Buckle, V. J. , & Higgs, D. R. (2022). On‐microscope staging of live cells reveals changes in the dynamics of transcriptional bursting during differentiation. Nature Communications, 13(1), 6641. 10.1038/s41467-022-33977-4 PMC9636426 36333299

[74] Oudelaar, A. M. , Davies, J. O. J. , Hanssen, L. L. P. , Telenius, J. M. , Schwessinger, R. , Liu, Y. , Brown, J. M. , Downes, D. J. , Chiariello, A. M. , Bianco, S. , Nicodemi, M. , Buckle, V. J. , Dekker, J. , Higgs, D. R. , & Hughes, J. R. (2018). Single‐allele chromatin interactions identify regulatory hubs in dynamic compartmentalized domains. Nature Genetics, 50(12), 1744–1751. 10.1038/s41588-018-0253-2 30374068 PMC6265079

[75] Quintero‐Cadena, P. , & Sternberg, P. W. (2016). Enhancer sharing promotes neighborhoods of transcriptional regulation across eukaryotes. G3 Genes|Genomes|Genetics, 6(12), 4167–4174. 10.1534/g3.116.036228 27799341 PMC5144984

[76] Fukaya, T. , Lim, B. , & Levine, M. (2016). Enhancer control of transcriptional bursting. Cell, 166(2), 358–368. 10.1016/j.cell.2016.05.025 27293191 PMC4970759

[77] Moorthy, S. D. , Davidson, S. , Shchuka, V. M. , Singh, G. , Malek‐Gilani, N. , Langroudi, L. , Martchenko, A. , So, V. , Macpherson, N. N. , & Mitchell, J. A. (2017). Enhancers and super‐enhancers have an equivalent regulatory role in embryonic stem cells through regulation of single or multiple genes. Genome Research, 27(2), 246–258. 10.1101/gr.210930.116 27895109 PMC5287230

[78] Miller, S. W. , & Posakony, J. W. (2020). Disparate expression specificities coded by a shared Hox‐C enhancer. ELife, 9, e39876. 10.7554/elife.39876 32342858 PMC7188484

[79] Abdella, R. , Talyzina, A. , Chen, S. , Inouye, C. J. , Tjian, R. , & He, Y. (2021). Structure of the human mediator‐bound transcription preinitiation complex. Science, 372(6537), 52–56. 10.1126/science.abg3074 33707221 PMC8117670

[80] Chen, X. , Yin, X. , Li, J. , Wu, Z. , Qi, Y. , Wang, X. , Liu, W. , & Xu, Y. (2021). Structures of the human mediator and mediator‐bound preinitiation complex. Science, 372(6546), eabg0635. 10.1126/science.abg0635 33958484

[81] Khattabi, L. E. , Zhao, H. , Kalchschmidt, J. , Young, N. , Jung, S. , Blerkom, P. V. , Kieffer‐Kwon, P. , Kieffer‐Kwon, K.‐R. , Park, S. , Wang, X. , Krebs, J. , Tripathi, S. , Sakabe, N. , Sobreira, D. R. , Huang, S.‐C. , Rao, S. S. P. , Pruett, N. , Chauss, D. , Sadler, E. , … Casellas, R. (2019). A pliable mediator acts as a functional rather than an architectural bridge between promoters and enhancers. Cell, 178(5), 1145–1158.e20. 10.1016/j.cell.2019.07.011 31402173 PMC7533040

[82] Sun, F. , Sun, T. , Kronenberg, M. , Tan, X. , Huang, C. , & Carey, M. F. (2021). The Pol II Preinitiation Complex (PIC) influences mediator binding but not promoter – enhancer looping. Genes & Development, 35(15–16), 1175–1189. 10.1101/gad.348471.121 34301767 PMC8336890

[83] Ramasamy, S. , Aljahani, A. , Karpinska, M. A. , Cao, T. B. N. , Cruz, J. N. , & Oudelaar, A. M. (2022). The mediator complex regulates enhancer‐promoter interactions. BioRxiv, 10.1101/2022.06.15.496245 PMC10352134 37430065

[84] Li, J. , Hsu, A. , Hua, Y. , Wang, G. , Cheng, L. , Ochiai, H. , Yamamoto, T. , & Pertsinidis, A. (2020). Single‐gene imaging links genome topology, promoter – enhancer communication and transcription control. Nature Structural & Molecular Biology, 27(11), 1032–1040. 10.1038/s41594-020-0493-6 PMC7644657 32958948

[85] Li, J. , & Pertsinidis, A. (2021). New insights into promoter – enhancer communication mechanisms revealed by dynamic single‐molecule imaging. Biochemical Society Transactions, 49(3), 1299–1309. 10.1042/bst20200963 34060610 PMC8325597

[86] Kowalczyk, M. S. , Hughes, J. R. , Garrick, D. , Lynch, M. D. , Sharpe, J. A. , Sloane‐Stanley, J. A. , McGowan, S. J. , De Gobbi, M. , Hosseini, M. , Vernimmen, D. , Brown, J. M. , Gray, N. E. , Collavin, L. , Gibbons, R. J. , Flint, J. , Taylor, S. , Buckle, V. J. , Milne, T. A. , Wood, W. G. , & Higgs, D. R. (2012). Intragenic enhancers act as alternative promoters. Molecular Cell, 45(4), 447–458. 10.1016/j.molcel.2011.12.021 22264824

[87] Li, G. , Ruan, X. , Auerbach, R. , Sandhu, K. , Zheng, M. , Wang, P. , Poh, H. , Goh, Y. , Lim, J. , Zhang, J. , Sim, H. , Peh, S. , Mulawadi, F. , Ong, C. , Orlov, Y. , Hong, S. , Zhang, Z. , Landt, S. , Raha, D. , … Ruan, Y. (2012). Extensive promoter‐centered chromatin interactions provide a topological basis for transcription regulation. Cell, 148(1–2), 84–98. 10.1016/j.cell.2011.12.014 22265404 PMC3339270

[88] Harrold, C. L. , Gosden, M. E. , Hanssen, L. L. P. , Stolper, R. J. , Downes, D. J. , Telenius, J. M. , Biggs, D. , Preece, C. , Alghadban, S. , Sharpe, J. A. , Davies, B. , Sloane‐Stanley, J. A. , Kassouf, M. T. , Hughes, J. R. , & Higgs, D. R. (2020). A functional overlap between actively transcribed genes and chromatin boundary elements. BioRxiv, 10.1101/2020.07.01.182089 PMC13083895 41840057

[89] Grosveld, F. , van Staalduinen, J. , & Stadhouders, R. (2021). Transcriptional regulation by (super)enhancers: From discovery to mechanisms. Annual Review of Genomics and Human Genetics, 22(1), 127–146. 10.1146/annurev-genom-122220-093818 33951408

[90] Banerji, J. , Olson, L. , & Schaffner, W. (1983). A lymphocyte‐specific cellular enhancer is located downstream of the joining region in immunoglobulin heavy chain genes. Cell, 33(3), 729–740. 10.1016/0092-8674(83)90015-6 6409418

[91] Banerji, J. , Rusconi, S. , & Schaffner, W. (1981). Expression of a β‐globin gene is enhanced by remote SV40 DNA sequences. Cell, 27(2), 299–308. 10.1016/0092-8674(81)90413-x 6277502

[92] Kassouf, M. T. , Francis, H. S. , Gosden, M. , Suciu, M. C. , Downes, D. J. , Harrold, C. , Larke, M. , Oudelaar, M. , Cornell, L. , Blayney, J. , Telenius, J. , Xella, B. , Shen, Y. , Sousos, N. , Sharpe, J. A. , Sloane‐Stanley, J. , Smith, A. , Babbs, C. , Hughes, J. R. , & Higgs, D. R. (2022). Multipartite super‐enhancers function in an orientation‐dependent manner. BioRxiv, 10.1101/2022.07.14.499999

[93] Canver, M. C. , Smith, E. C. , Sher, F. , Pinello, L. , Sanjana, N. E. , Shalem, O. , Chen, D. D. , Schupp, P. G. , Vinjamur, D. S. , Garcia, S. P. , Luc, S. , Kurita, R. , Nakamura, Y. , Fujiwara, Y. , Maeda, T. , Yuan, G.‐C. , Feng, Z. , Orkin, S. H. , & Bauer, D. E. (2015). BCL11A enhancer dissection by Cas9‐mediated in situ saturating mutagenesis. Nature, 527(7577), 192–197. 10.1038/nature15521 26375006 PMC4644101

[94] Mettananda, S. , Fisher, C. A. , Hay, D. , Badat, M. , Quek, L. , Clark, K. , Hublitz, P. , Downes, D. , Kerry, J. , Gosden, M. , Telenius, J. , Sloane‐Stanley, J. A. , Faustino, P. , Coelho, A. , Doondeea, J. , Usukhbayar, B. , Sopp, P. , Sharpe, J. A. , Hughes, J. R. , … Higgs, D. R. (2017). Editing an α‐globin enhancer in primary human hematopoietic stem cells as a treatment for β‐thalassemia. Nature Communications, 8(1), 424. 10.1038/s41467-017-00479-7 PMC5583283 28871148

[95] Vian, L. , Pękowska, A. , Rao, S. S. P. , Kieffer‐Kwon, K.‐R. , Jung, S. , Baranello, L. , Huang, S.‐C. , Khattabi, L. E. , Dose, M. , Pruett, N. , Sanborn, A. L. , Canela, A. , Maman, Y. , Oksanen, A. , Resch, W. , Li, X. , Lee, B. , Kovalchuk, A. L. , Tang, Z. , … Casellas, R. (2018). The energetics and physiological impact of cohesin extrusion. Cell, 173(5), 1165–1178.e20. 10.1016/j.cell.2018.03.072 29706548 PMC6065110

[96] Barrington, C. , Georgopoulou, D. , Pezic, D. , Varsally, W. , Herrero, J. , & Hadjur, S. (2019). Enhancer accessibility and CTCF occupancy underlie asymmetric TAD architecture and cell type specific genome topology. Nature Communications, 10(1), 2908. 10.1038/s41467-019-10725-9 PMC6606583 31266948

[97] Hsieh, T.‐H. S. , Cattoglio, C. , Slobodyanyuk, E. , Hansen, A. S. , Rando, O. J. , Tjian, R. , & Darzacq, X. (2020). Resolving the 3D landscape of transcription‐linked mammalian chromatin folding. Molecular Cell, 78(3), 539–553.e8. 10.1016/j.molcel.2020.03.002 32213323 PMC7703524

[98] Tanimoto, K. , Liu, Q. , Bungert, J. , & Engel, J. D. (1999). Effects of altered gene order or orientation of the locus control region on human β‐globin gene expression in mice. Nature, 398(6725), 344–348. 10.1038/18698 10192336

[99] Noordermeer, D. , Branco, M. R. , Splinter, E. , Klous, P. , van IJcken, W. , Swagemakers, S. , Koutsourakis, M. , van der Spek, P. , Pombo, A. , & de Laat, W. (2008). Transcription and chromatin organization of a housekeeping gene cluster containing an integrated β‐globin locus control region. PLoS Genetics, 4(3), e1000016. 10.1371/journal.pgen.1000016 18369441 PMC2265466

[100] Despang, A. , Schöpflin, R. , Franke, M. , Ali, S. , Jerković, I. , Paliou, C. , Chan, W.‐L. , Timmermann, B. , Wittler, L. , Vingron, M. , Mundlos, S. , & Ibrahim, D. M. (2019). Functional dissection of the Sox9 – Kcnj2 locus identifies nonessential and instructive roles of TAD architecture. Nature Genetics, 51(8), 1263–1271. 10.1038/s41588-019-0466-z 31358994

[101] Maurano, M. T. , Humbert, R. , Rynes, E. , Thurman, R. E. , Haugen, E. , Wang, H. , Reynolds, A. P. , Sandstrom, R. , Qu, H. , Brody, J. , Shafer, A. , Neri, F. , Lee, K. , Kutyavin, T. , Stehling‐Sun, S. , Johnson, A. K. , Canfield, T. K. , Giste, E. , Diegel, M. , … Stamatoyannopoulos, J. A. (2012). Systematic localization of common disease‐associated variation in regulatory DNA. Science, 337(6099), 1190–1195. 10.1126/science.1222794 22955828 PMC3771521

[102] Vierstra, J. , Lazar, J. , Sandstrom, R. , Halow, J. , Lee, K. , Bates, D. , Diegel, M. , Dunn, D. , Neri, F. , Haugen, E. , Rynes, E. , Reynolds, A. , Nelson, J. , Johnson, A. , Frerker, M. , Buckley, M. , Kaul, R. , Meuleman, W. , & Stamatoyannopoulos, J. A. (2020). Global reference mapping of human transcription factor footprints. Nature, 583(7818), 729–736. 10.1038/s41586-020-2528-x 32728250 PMC7410829

[103] Meuleman, W. , Muratov, A. , Rynes, E. , Halow, J. , Lee, K. , Bates, D. , Diegel, M. , Dunn, D. , Neri, F. , Teodosiadis, A. , Reynolds, A. , Haugen, E. , Nelson, J. , Johnson, A. , Frerker, M. , Buckley, M. , Sandstrom, R. , Vierstra, J. , Kaul, R. , & Stamatoyannopoulos, J. (2020). Index and biological spectrum of human DNase I hypersensitive sites. Nature, 584(7820), 244–251. 10.1038/s41586-020-2559-3 32728217 PMC7422677

[104] Javierre, B. M. , Burren, O. S. , Wilder, S. P. , Kreuzhuber, R. , Hill, S. M. , Sewitz, S. , Cairns, J. , Wingett, S. W. , Várnai, C. , Thiecke, M. J. , Burden, F. , Farrow, S. , Cutler, A. J. , Rehnström, K. , Downes, K. , Grassi, L. , Kostadima, M. , Freire‐Pritchett, P. , Wang, F. , … Fraser, P. (2016). Lineage‐specific genome architecture links enhancers and non‐coding disease variants to target gene promoters. Cell, 167(5), 1369–1384.e19. 10.1016/j.cell.2016.09.037 27863249 PMC5123897

[105] Zhu, Z. , Zhang, F. , Hu, H. , Bakshi, A. , Robinson, M. R. , Powell, J. E. , Montgomery, G. W. , Goddard, M. E. , Wray, N. R. , Visscher, P. M. , & Yang, J. (2016). Integration of summary data from GWAS and eQTL studies predicts complex trait gene targets. Nature Genetics, 48(5), 481–487. 10.1038/ng.3538 27019110

[106] Downes, D. J. , Cross, A. R. , Hua, P. , Roberts, N. , Schwessinger, R. , Cutler, A. J. , Munis, A. M. , Brown, J. , Mielczarek, O. , de Andrea, C. E. , Melero, I. , Gill, D. R. , Hyde, S. C. , Knight, J. C. , Todd, J. A. , Sansom, S. N. , Issa, F. , Davies, J. O. J. , & Hughes, J. R. , Consortium, Co.‐19 M. B. At. (COMBAT) . (2021). Identification of LZTFL1 as a candidate effector gene at a COVID‐19 risk locus. Nature Genetics, 53(11), 1606–1615. 10.1038/s41588-021-00955-3 34737427 PMC7611960

[107] Ng, S. B. , Turner, E. H. , Robertson, P. D. , Flygare, S. D. , Bigham, A. W. , Lee, C. , Shaffer, T. , Wong, M. , Bhattacharjee, A. , Eichler, E. E. , Bamshad, M. , Nickerson, D. A. , & Shendure, J. (2009). Targeted capture and massively parallel sequencing of 12 human exomes. Nature, 461(7261), 272–276. 10.1038/nature08250 19684571 PMC2844771

[108] Spielmann, M. , Lupiáñez, D. G. , & Mundlos, S. (2018). Structural variation in the 3D genome. Nature Reviews Genetics, 19(7), 453–467. 10.1038/s41576-018-0007-0 29692413

[109] Smith, E. , & Shilatifard, A. (2014). Enhancer biology and enhanceropathies. Nature Structural & Molecular Biology, 21(3), 210–219. 10.1038/nsmb.2784 24599251

[110] Zaugg, J. B. , Sahlén, P. , Andersson, R. , Alberich‐Jorda, M. , de Laat, W. , Deplancke, B. , Ferrer, J. , Mandrup, S. , Natoli, G. , Plewczynski, D. , Rada‐Iglesias, A. , & Spicuglia, S. (2022). Current challenges in understanding the role of enhancers in disease. Nature Structural & Molecular Biology, 29(12), 1148–1158. 10.1038/s41594-022-00896-3 36482255

[111] Badat, M. , Davies, J. O. J. , Fisher, C. A. , Downes, D. J. , Rose, A. , Glenthøj, A. B. , van Beers, E. J. , Harteveld, C. L. , & Higgs, D. R. (2021). A remarkable case of HbH disease illustrates the relative contributions of the α‐globin enhancers to gene expression. Blood, 137(4), 572–575. 10.1182/blood.2020006680 33113553

